# *Nicotiana benthamiana* phosphatidylinositol 4‐kinase type II regulates chilli leaf curl virus pathogenesis

**DOI:** 10.1111/mpp.12846

**Published:** 2019-09-02

**Authors:** Mansi, Nirbhay Kumar Kushwaha, Ashish Kumar Singh, Mir Jishan Karim, Supriya Chakraborty

**Affiliations:** ^1^ Molecular Virology Laboratory, School of Life Sciences Jawaharlal Nehru University New Delhi India

**Keywords:** geminivirus, kinase, pathogenesis, PI4K, Rep protein

## Abstract

Geminiviruses are single‐stranded DNA viruses that can cause significant losses in economically important crops. In recent years, the role of different kinases in geminivirus pathogenesis has been emphasized. Although geminiviruses use several host kinases, the role of phosphatidylinositol 4‐kinase (PI4K) remains obscure. We isolated and characterized phosphatidylinositol 4‐kinase type II from *Nicotiana benthamiana* (NbPI4KII) which interacts with the replication initiator protein (Rep) of a geminivirus, chilli leaf curl virus (ChiLCV). NbPI4KII‐mGFP was localized into cytoplasm, nucleus or both. NbPI4KII‐mGFP was also found to be associated with the cytoplasmic endomembrane systems in the presence of ChiLCV. Furthermore, we demonstrated that Rep protein directly interacts with NbPI4KII protein and influenced nuclear occurrence of NbPI4KII. The results obtained in the present study revealed that NbPI4KII is a functional protein kinase lacking lipid kinase activity. Downregulation of *NbPI4KII* expression negatively affects ChiLCV pathogenesis in *N. benthamiana*. In summary, NbPI4KII is a susceptible factor, which is required by ChiLCV for pathogenesis.

## Introduction

Phosphatidylinositol 4‐kinase (PI4K) is an enzyme responsible for catalysing the first committed step in the biosynthesis of the phosphatidylinositols (PtdIns) PtdIns (4,5)P_2_ and PtdIns (1,3,5)P_3_, two important signalling molecules (Carpenter and Cantley, 1996; Toker and Cantley, 1997; Fruman *et al*., 1998; Cantrell, 2001; Cockcroft, 2001; Cantley, 2002). PI4Ks catalyse phosphorylation of the inositol ring of inositol phospholipids in the fourth OH position. PI4Ks are categorized into two types based on their catalytic properties and sensitivity to PI3K inhibitor. Type II PI4Ks are insensitive to wortmannin, unlike type III, a property that places type III PI4K close to PI3K. While the plant type III PI4Ks have been extensively studied, little is known about role of the type II PI4Ks.

Geminiviruses constitute the largest group of plant viruses that are recognized for detrimental losses of economically important crops like tomato, cotton, cassava, maize and chickpea (Moffat, 1999). Chilli leaf curl virus (ChiLCV) is a ssDNA‐containing monopartite virus, species *Chilli leaf curl virus*, genus *Begomovirus*, family *Geminiviridae,* that causes chilli leaf curl disease (ChiLCD), which is characterized by leaf curling, leaf distortion, crinkling of leaves and stunting of plants, and results in significant yield loss (Chattopadhyay *et al*., 2008; Kumar *et al*., 2015).

Like other obligate parasites, geminiviruses also depend on several host factors for successful pathogenesis in a permissive host. The interplay between geminivirus and host kinases plays a crucial role in geminivirus replication, expression and pathogenesis (Hanley‐Bowdoin *et al*., 2013). Kinases are known to alter the activity of substrate by transferring terminal phosphate from adenosine triphosphate (ATP) to its substrate. Phosphorylation of viral‐encoded proteins is known to influence pathogenesis (Shen *et al*., 2011). In some cases, phosphorylation of viral proteins leads to attenuation of infection, whereas in others it results in potentiation of viral pathogenesis. In one of the studies, *Solanum lycopersicum* SUCROSE‐NON FERMENTING1‐related kinase (SlSnRK1) interacts with and phosphorylates the tomato yellow leaf curl China virus (TYLCCNV)‐encoded βC1 protein, resulting in attenuation of geminivirus infection (Shen *et al*., 2011). It was found that the C4 protein of beet curly top virus (BCTV) is phosphorylated at the serine 49 residue and activated by shaggy‐like kinase, consequently interfering with the brassinosteroid signalling pathway of the host (Piroux *et al*., 2007). A proline‐rich extensin‐like receptor protein kinase (PERK) interacts with nuclear shuttle protein of cabbage leaf curl virus (CaLCuV). The interaction results in phosphorylation of nuclear shuttle protein (NSP), which positively regulates CaLCuV (Florentino *et al*., 2006). It is also observed that kinases can affect viral pathogenesis indirectly without phosphorylation. *Solanum lycopersicon* kinase and its homologue in *Glycine max* NSP‐interacting kinase SlNIK1 and GmNIK1, respectively, were found to interact with NSP of tomato golden mosaic virus (TGMV) and tomato crinkle leaf yellow virus (TCrLYV) (Mariano *et al*., 2004). The interaction between NSP and  NSP‐interacting kinase I (NIK I) is implicated in suppression of NIK1‐mediated antiviral defence (Carvalho *et al*., 2008).

Phosphorylation of viral proteins can also regulate the movement of plant viruses. For example, phosphorylation of the P30 movement protein (MP) of tomato mosaic virus (ToMV) by protein kinase C (PKC) is crucial for its intracellular movement and efficient spread of virus (Kawakami *et al*., 1999). Likewise, the successful establishment of symptoms by potato leaf roll virus (PLRV) is achieved through phosphorylation of pr17 protein (Sokolova *et al*., 1997). Similarly, turnip yellow mosaic virus (TYMV) 69K MP phosphorylation is important for symptom development and viral pathogenesis (Seron *et al*., 1996).

The role of PI4K in the pathogenesis of RNA viruses infecting animals is well established. PI4KIIIα is well known to favour hepatitis C virus (HCV) replication. PI4KIIIα interacts with and phosphorylates non‐structural protein 5A (NS5A), resulting in activation of the enzyme thereby producing more phosphatidylinositol 4‐phosphate, which acts as replication hub for HCV (Berger *et al*., 2011; Reiss *et al*., 2011). However, no role for PI4K has been reported to date in the case of plant viruses. The current study, for the first time, highlights the role of PI4KII in geminivirus pathogenesis. Here, we demonstrate that NbPI4KII interacts with the ChiLCV Rep protein and positively regulates ChiLCV pathogenesis.

## Results

### Isolation and phylogenetic analysis of *PI4KII*

**Figure 1:**
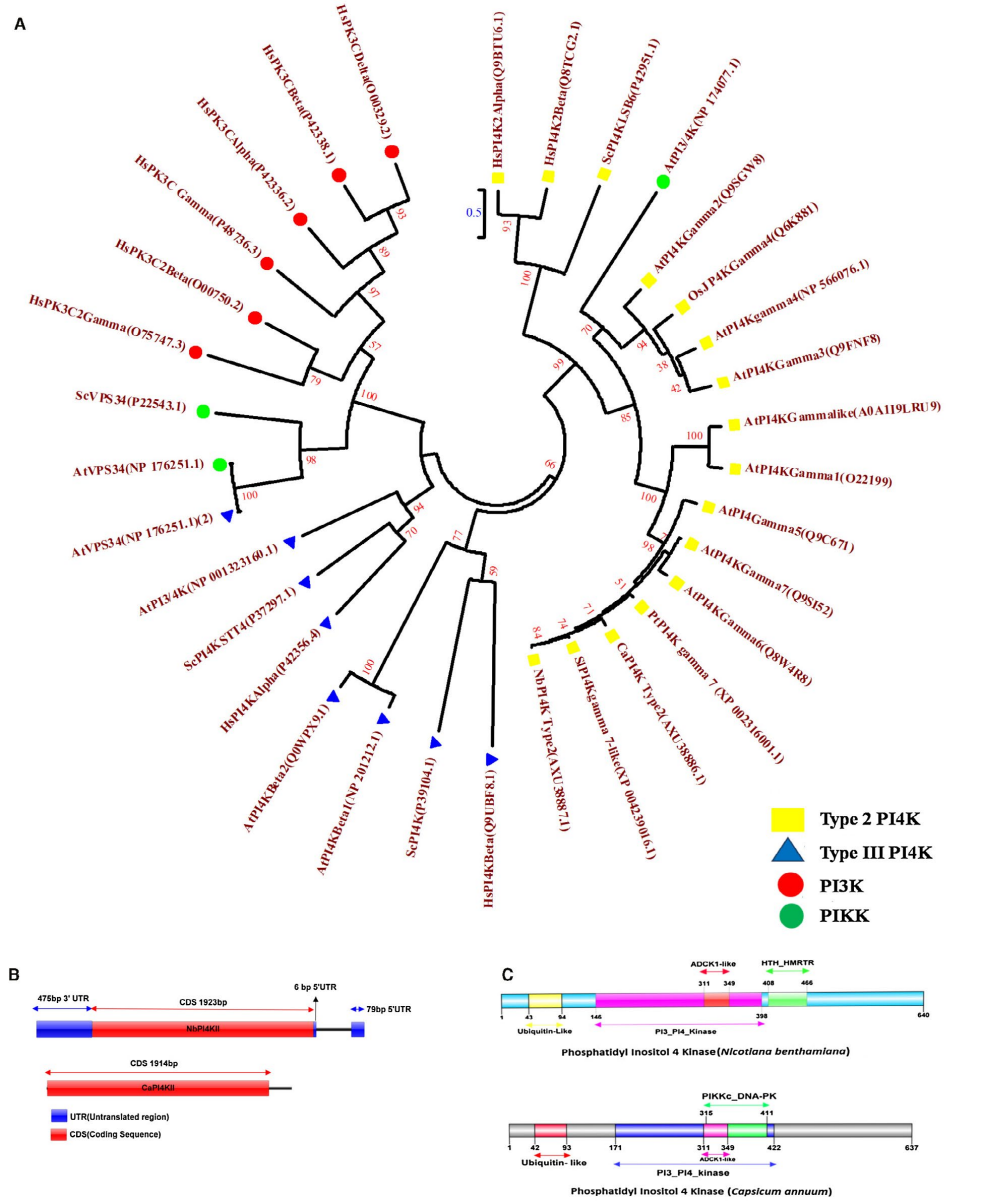
(A) Phylogenetic analysis of various PI4Ks using the maximum likelihood method. Each yellow rectangle denotes type II PI4K, a blue triangle indicates type III PI4K, while red and green circles are for PI3K and PIKK, respectively. Protein names and their corresponding accession numbers are also indicated in the tree. (B) Schematic diagram of NbPI4K and CaPI4K genes. CDS is shown in red and UTRs are presented in blue. (C) Domain architecture of NbPI4KII and CaPI4KII. NbPI4K type II domains‐ phosphatidylinositol 3‐ and 4‐kinase domain (pink), aarF domain containing kinase 1 domain (red), helix‐turn‐helix DNA‐binding domain of heavy metal resistance transcription regulators (green) and ubiquitin‐like domain (yellow). CaPI4K type II domains containing phosphatidylinositol 3‐ and 4‐kinase domain (blue), aarF domain containing kinase 1 domain (pink), catalytic domain of DNA‐dependent protein kinase (green) and ubiquitin‐like domain (red).

In a previous study, PI4K was differentially expressed in *Capsicum annuum* plants following ChiLCV infection (Kushwaha *et al*., 2015). Furthermore, to study the effect of ChiLCV on *PI4KII* expression, the transcript level of *PI4KII* was assessed in ChiLCV‐ and mock‐infiltrated *Nicotiana benthamiana* and *C. annuum* plants at different days post‐inoculation (dpi). Total RNA was isolated from both the plants at 7, 14 and 21 dpi, and cDNA was prepared to analyse the *PI4KII* expression level by quantitative real‐time PCR (qRT‐PCR) using *PI4KII*‐specific primer pairs. Both the *NbPI4KII* and*CaPI4KII* transcript levels were increased significantly following ChiLCV infection (Fig. S1A,B). In both cases, mock‐treated plants showed a comparatively reduced level of *PI4KII* expression. We therefore investigated the role of PI4K in ChiLCV pathogenesis. *PI4KII* was isolated from susceptible model plant *N. benthamiana* (1923 nt) and *C. annuum* 'Punjab lal' (1914 nt). The phylogenetic analysis of NbPI4K and CaPI4K was carried out using MEGA 7.0 with 1000 bootstrap replicates (Kumar *et al*., 2016). Phylogenetic analysis derived from the alignment of the deduced amino acid sequences of PI4K from *Homo sapiens*, *Saccharomyces cerevisiae*, *Arabidopsis thaliana*, *S. lycopersicum*, *Oryza sativa*, *C. annuum* and *N. benthamiana* revealed that the isolated homologue from *N. benthamiana* and *C. annum* belongs to PI4K type II clade (Fig. 1A). Furthermore, amino acid sequences of PI4KII of these organisms were aligned by ClustalW. NbPI4KII shares maximum identity (91.4%) with SlPI4KII Gamma7 (GenBank accession AXU38886) and minimum identity (12.8%) with HsPI4K2α (*H. sapiens*) (GenBank accession no. Q9BTU6), whereas CaPI4KII possesses maximum identity of 81.9% with SlPI4KII Gamma7 and minimum identity of 16.1% with HsPI4K2α (Supplementary Table S1). NbPI4KII shares 81.8% identity with CaPI4KII.

To determine the locus structure, we applied BLAST to the *NbPI4KII* sequence on the *N. benthamiana* genome sequence available in the Sol Genomics Network database (https://solgenomics.net). *NbPI4KII* with 1923 nts was observed to span from nt number 1422 to nt number 3335 of the (Niben101Scf04187Ctg023) and *NbPI4KII* was found to be organized into one exon and two introns (one 475 bp 3′UTR and two 5′UTR of 6 and 79 bp) (Fig. 1B). Similarly, we have also used BLAST analysis to identify the locus structure of the *CaPI4KII* sequence on the *C. annuum* genome sequence available on the Sol Genomics database and it was observed that *CaPI4KII* with an intronless coding region of 1914 nt spans nt number 122817209 to nt number 122819078 of the chromosome number 5 of *C. annum* UCD10X genome chromosome (v.1.0) (Fig. 1B).

*NbPI4KII* encodes a 640 amino acid protein having a phosphatidylinositol 3‐ and 4‐kinase domain (protein kinase) that also contains an aarF domain containing a kinase 1 domain (Fig. 1C). Furthermore, the Motif finder result showed the presence of a helix‐turn‐helix DNA‐binding domain of heavy metal resistance transcription regulators and a ubiquitin‐like domain in NbPI4KII. On the other hand, CaPI4K type 2 protein comprises 637 amino acids, NCBI‐CDD, and Motif finder scan revealed that it comprised one phosphatidylinositol 3‐ and 4‐kinase domain between 171 and 422 amino acids region in addition to aarF domain containing kinase 1 domain and catalytic domain of DNA‐dependent protein kinase (Fig. 1C). The Motif finder tool revealed that CaPI4KII consisted of a ubiquitin‐like domain at the 42 to 93 amino acid region.

### NbPI4KII and CaPI4KII interact with ChiLCV proteins

**Figure 2:**
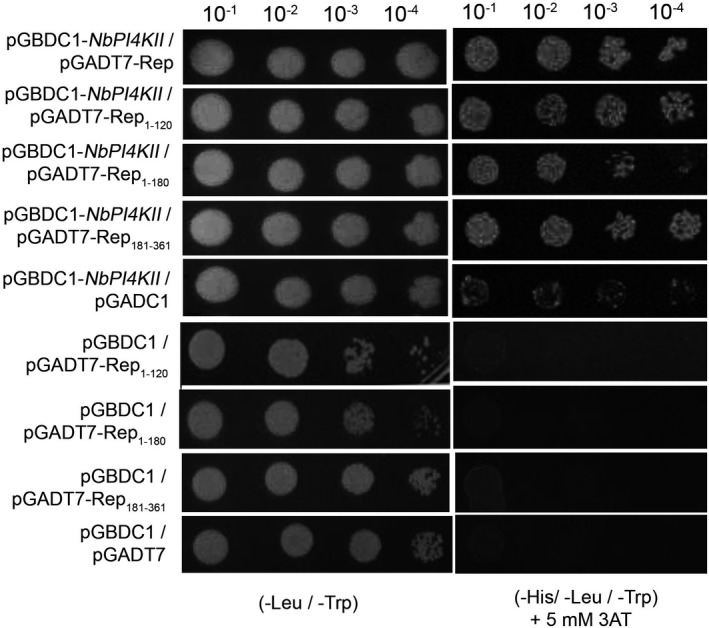
ChiLCV Rep protein interacts with NbPI4KII. Yeast two‐hybrid assays of NbPI4KII with either Rep or Rep domains on nonselective media (‐Leu, ‐Trp) and selective media (‐His, ‐Leu, ‐Trp with 5 mM 3AT).

**Figure 3:**
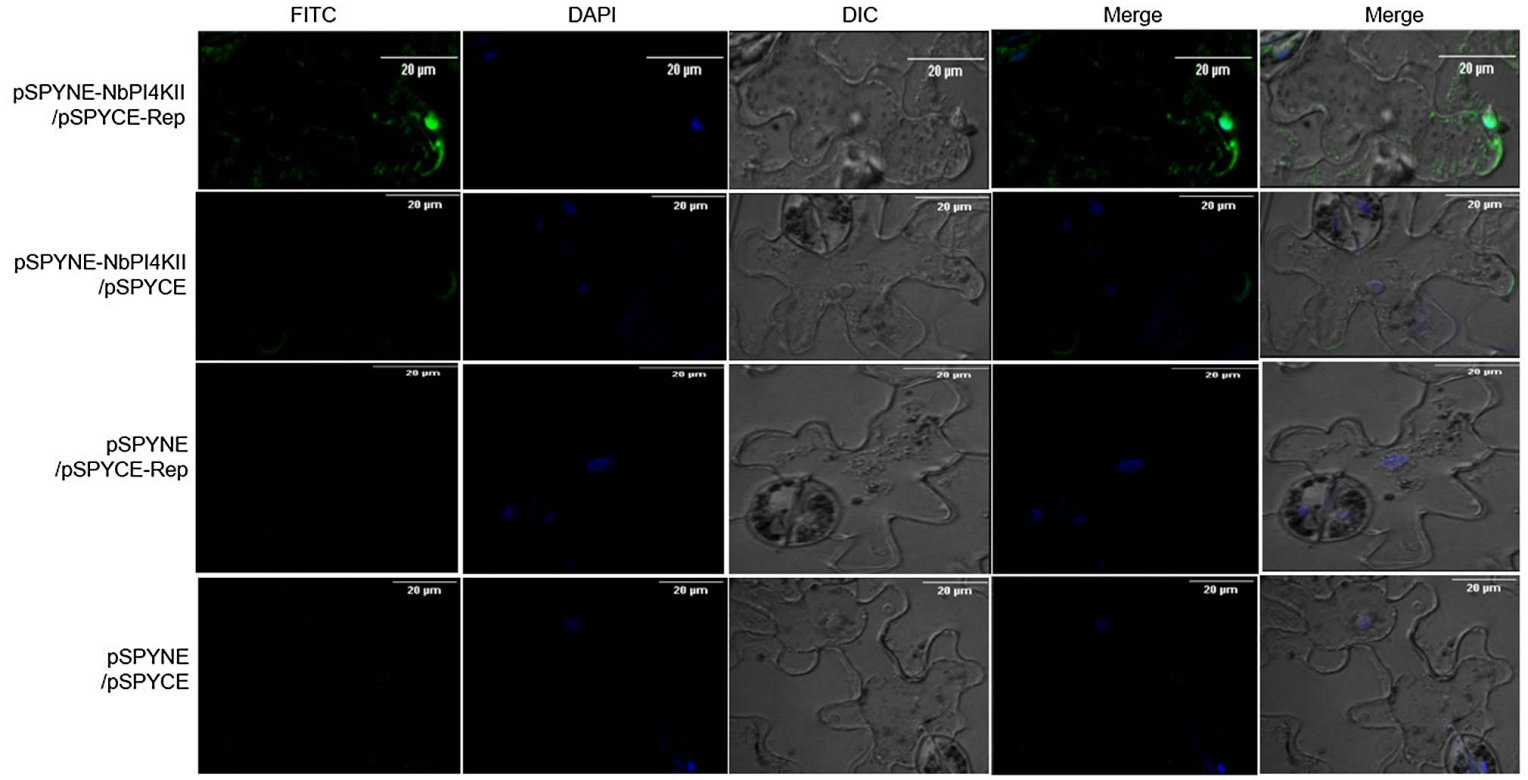
NbPI4KII interacts with ChiLCV Rep *in vivo*. *In planta* bimolecular fluorescence complementation assays were performed in the lower epidermis of *Nicotiana benthamiana* leaves (at 8 days post‐infiltration). NbPI4KII was expressed as in‐frame fusion with the N‐terminal of the YFP protein using the pSPYNE vector. ChiLCV Rep protein was expressed with C‐terminal region of the YFP protein using pSPYCE vector (Kushwaha *et al*., 2017). Bimolecular fluorescence complementation (BiFC) assays of interaction of (A) pSPYNE‐NbPI4KII and pSPYCE‐Rep, (B) pSPYNE‐ NbPI4KII and pSPYCE, (C) pSPYNE and pSPYCE‐Rep and (D) pSPYNE / pSPYCE. Scale bar = 20 μm.

To identify NbPI4KII‐ and CaPI4KII‐interacting ChiLCV proteins, protein–protein interaction studies were performed using yeast two‐hybrid assays. We identified four interacting partners of NbPI4KII: replication initiator protein (Rep) (Fig. 2), transactivator protein (TrAP), pre‐coat protein (V2) and C4 proteins (Fig. S2A). Since Rep is a multifunctional protein and is indispensable for viral pathogenesis, we focused our further study on Rep. In addition, a bimolecular fluorescence complementation (BiFC) assay was conducted on the *N. benthamiana* leaves by co‐infiltration of pSPYCE‐Rep and pSPYNE‐NbPI4KII constructs through agroinfiltration. Combinations of pSPYCE‐Rep–pSPYNE (empty vector), pSPYCE (empty vector)–pSPYNE‐NbPI4KII, pSPYCE (empty vector)–pSPYNE (empty vector) served as control. At 8 days post‐infiltration, the samples were observed under a confocal microscope (Model Eclipse TiE, Nikon, Tokyo, Japan) by using an FITC filter. The BiFC assay showed a positive interaction between NbPI4KII and ChiLCV Rep protein, whereas leaves agroinfiltrated with control combinations did not yield any signal (Fig. 3).

Rep has different domains contributing to different functions, therefore we wanted to study which domains of Rep (Rep_1‐120_, Rep_1‐180_ and Rep_181‐361_) are responsible for interaction with NbPI4KII. Furthermore, we performed yeast two‐hybrid assays of NbPI4KII with different domains of Rep. We found that NbPI4KII interacts with all domains of Rep, i.e. Rep_1‐120_, Rep_1‐180_, and Rep_181‐361 _(Fig. 2). However, growth kinetics study revealed that interaction between full‐length Rep protein and NbPI4KII is weaker as compared to interactions between Rep domains and NbPI4KII as well as between pGADT7 and NbPI4KII (Fig. S2B). Interestingly, CaPI4KII failed to interact with the full‐length Rep protein, but it showed interaction with Rep domains Rep_1‐180_ and Rep_181‐361 _but not with the N‐terminal Rep_1‐120_ (Fig. S3).

### NbPI4KII interacts with Rep

**Figure 4:**
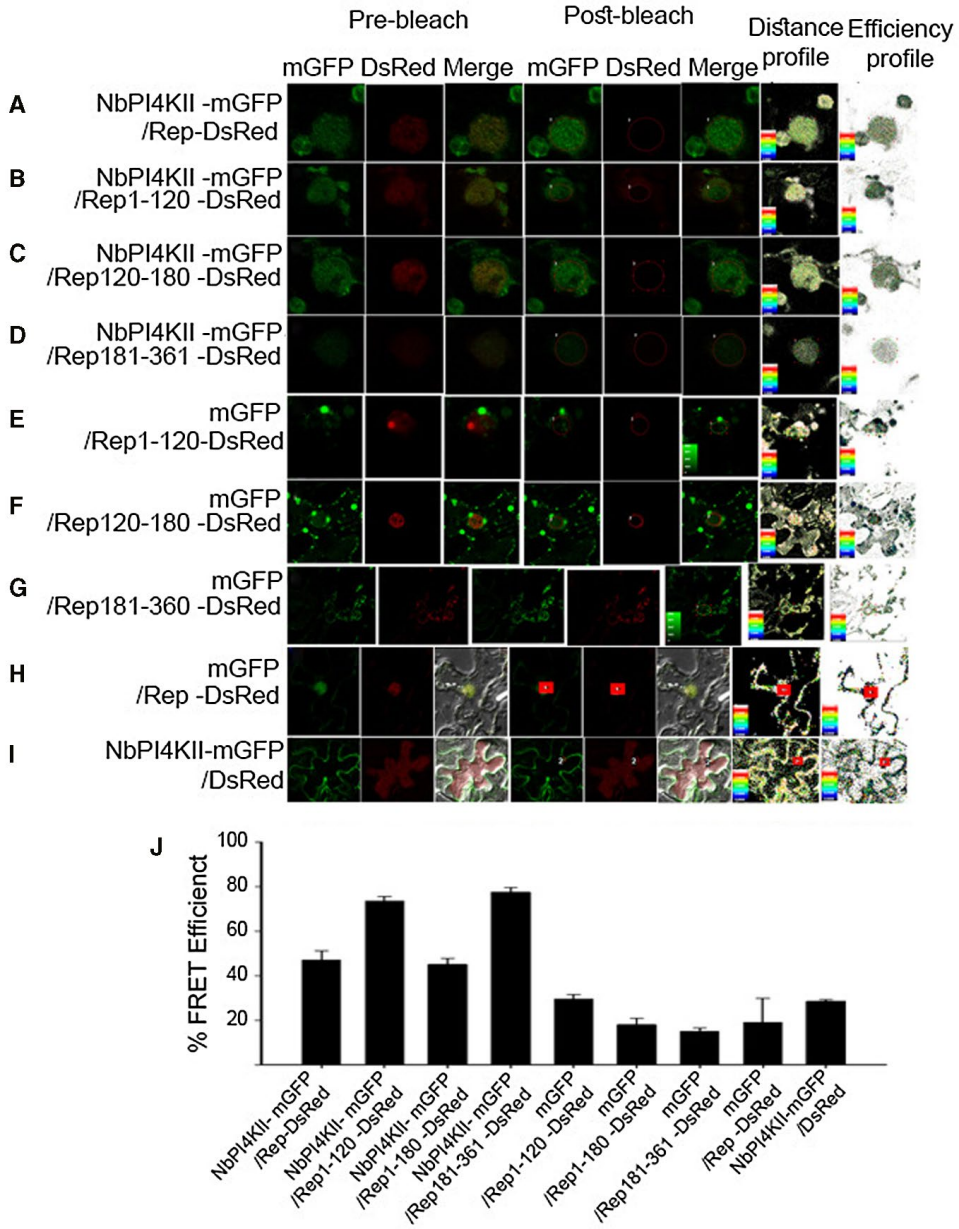
NbPI4KII directly interacts with Rep. Fluorescence resonance energy transfer (FRET) of NbPI4KII‐mGFP with (A) Rep‐DsRed, (B) Rep_1‐120_‐DsRed, (C) Rep_1‐180_‐DsRed and (D) Rep_181‐361_‐DsRed. FRET of mGFP with (E) Rep_1‐120_‐DsRed, (F) Rep_1‐180_‐DsRed, (G) Rep_181‐361_‐DsRed and (H) Rep‐DsRed. PI4KII‐mGFP and DsRed (I) also serves as control. Graphical depiction of percentage of FRET efficiency between different pairs used in this study. NbPI4KII‐mGFP acts as emitter and either Rep‐DsRed or its mutants acts as acceptor.

NbPI4KII interacts with ChiLCV Rep in yeast two‐hybrid assay. Also, NbPI4KII interacts with N‐terminal (Rep_1‐120_ and Rep_1‐180_) as well as C‐terminal domains (Rep_181‐360_) of Rep. To further validate the interaction *in planta*, fluorescence resonance energy transfer (FRET) assay was performed between NbPI4KII‐mGFP and Rep‐DsRed or truncated proteins of Rep, i.e. Rep_1‐120_‐DsRed, Rep_1‐180_‐DsRed or Rep_181‐361_‐DsRed. NbPI4KII‐mGFP was co‐expressed with either Rep‐DsRed or its truncated proteins in the *N. benthamiana* leaf epidermal cells. NbPI4KII‐mGFP showed 47% FRET efficiency with Rep‐DsRed (Figs 4A,J). Also, NbPI4KII‐mGFP showed efficiency of 72% with Rep_1‐120_‐DsRed, 43% with Rep_1‐180_‐DsRed and 76% with Rep_181‐361_‐DsRed (Fig. 4J). FRET between mGFP and Rep‐DsRed, Rep_1‐120_‐DsRed, Rep_1‐180_‐DsRed or Rep_181‐361_‐DsRed was performed as a negative control and showed efficiencies of 19%, 31%, 19% and 16%, respectively (Fig. 4E–J). In addition, NbPI4KII‐mGFP and DsRed showed FRET efficiency of 28% and also served as negative control (Fig. 4I,J).

### NbPI4KII is localized both in the cytoplasm and the nucleus

**Figure 5:**
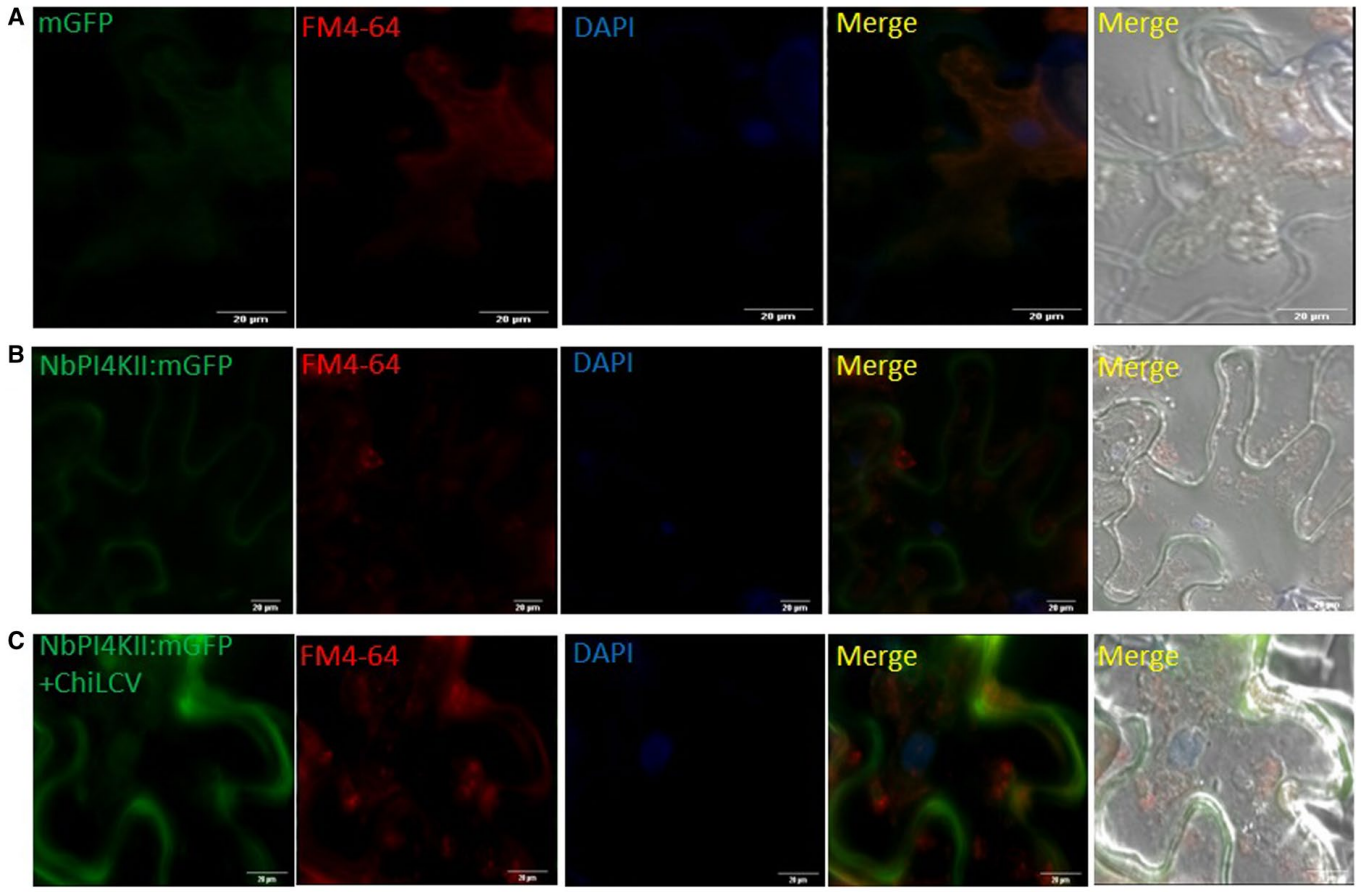
Subcellular localization of NbPI4KII‐mGFP in *Nicotiana benthamiana* leaf epidermal cells. (A) Subcellular localization of pCAMBIA 1304 expressing mGFP, (B) NbPI4KII‐mGFP and (C) NbPI4KII‐mGFP in presence of ChiLCV. DAPI is used to stain nucleus and FM4‐64 is used as both membrane and cytoplasmic marker. Scale bar = 20 µm.

Localization of NbPI4KII was studied by expressing protein in fusion with mGFP at the C‐terminus in the lower  epidermis of *N. benthamiana* leaves. Only mGFP expressed through pCAMBIA1304 serves as a control and the signal of mGFP was found throughout the cell (Fig. 5A). The subcellular localization study revealed that NbPI4KII‐mGFP is localized in the cytoplasm and the nucleus (Fig. 5B). NbPI4KII was observed in three states: in the cytoplasm and nucleus, only in the cytoplasm, and only in the nucleus. Notably, the percentage of cells showing NbPI4KII‐mGFP in both nucleus and cytoplasm was a maximum (67%) (Fig. S4A). Nuclear localization of NbPI4KII‐mGFP was confirmed by 4′,6‐diamidino‐2‐phenylindole (DAPI) staining (Fig. 5B). To further dissect the cytoplasmic location of NbPI4KII‐mGFP, the lower epidermal peel transiently expressing NbPI4KII‐mGFP was treated with *N*‐(3‐triethylammoniumpropyl)‐4‐(6‐(4‐(diethylamino) phenyl) hexatrienyl) pyridinium dibromide (FM4‐64). NbPI4KII‐mGFP was shown to be present at the plasma membranes as the signal of NbPI4KII‐mGFP merged with that of the signal of FM4‐64 (Fig. 5B). To study the effect of ChiLCV on the localization of NbPI4KII‐mGFP, we performed localization NbPI4KII in the presence of ChiLCV and expressed NbPI4KII‐mGFP in ChiLCV‐infected plants. The localization study revealed NbPI4KII‐mGFP signals were not restricted to the membrane only, but in the presence of ChiLCV NbPI4KII was found to be localized throughout the cytoplasm (Fig. 5C).

Furthermore, we also studied the localization of CaPI4KII‐mGFP and found that CaPI4KII‐mGFP, like NbPI4KII‐mGFP, localizes to the plasma membrane, the cytoplasm and the nucleus (Fig. S5A). The membrane and cytoplasmic occurrence was confirmed by FM4‐64 and nuclear occurrence by staining with DAPI (Fig. S5A). The percentage of cells showing CaPI4KII‐mGFP expression in the cytoplasm and nucleus was 79%, whereas that in the cytoplasm was 7% and in the nucleus 14% (Fig. S5B). Thus, the maximum fraction of cells, i.e. 55%, shows expression inside both the cytoplasm and nucleus (Fig. S5B). Similarly, CaPI4KII expression was also found to alter and was observed throughout the cell in the presence of ChiLCV (Fig. S6). FM4‐64 staining, when stained for longer duration, can stain cytoplasmic organelles like endoplasmic reticulum (ER) and Golgi bodies (GB). The FM4‐64 staining suggested the association of NbPI4KII‐mGFP and CaPI4KII‐mGFP with ER and GB in the cytoplasm (Fig. S6). These results suggest that association of PI4KII with subcellular organelles increases in the presence of ChiLCV.

### ChiLCV Rep protein mediates enhanced nuclear occurrence of NbPI4KII

**Figure 6:**
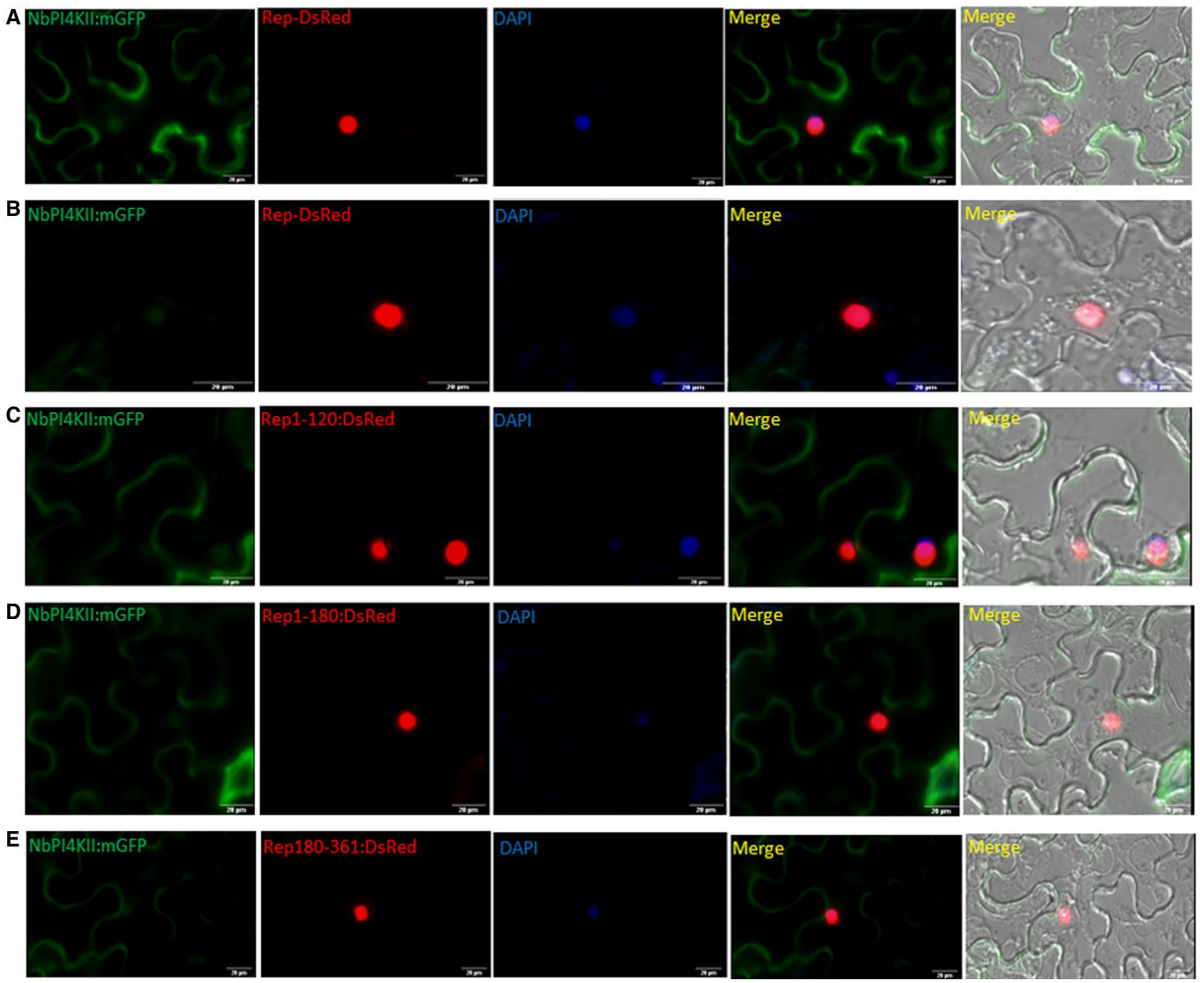
Colocalization study of NbPI4K‐mGFP with Rep‐DsRed and its mutants. Localization of NbPI4KII:mGFP with (A,B) Rep‐DsRed, (C) Rep_1‐120_‐DsRed, (D) Rep_1‐180_‐DsRed and (E) Rep_181‐361_‐DsRed. Scale bar = 20 µm.

To study the effect of ChiLCV Rep protein on the subcellular localization of NbPI4KII or vice versa, a co‐localization study was performed by co‐expressing *NbPI4KII*‐mGFP and 35S:*C1*‐*DsRed*. Nuclear localization of ChiLCV Rep‐DsRed has already been reported (Kushwaha *et al*., 2017). The subcellular localization study revealed that Rep‐DsRed was confined to the nucleus in the presence of NbPI4KII‐mGFP. NbPI4KII‐mGFP did not alter the Rep‐DsRed localization (Fig. 6A). NbPI4KII‐mGFP was observed to be present in two states, i.e. only inside the nucleus (63%), and in both cytoplasm and nucleus (37%), whereas no cells were found with expression restricted exclusively to the cytoplasm (*N* = 19) (Fig. 6A,B,F). It is noteworthy that the percentage of cells showing NbPI4KII‐mGFP only inside the nucleus in the presence of Rep‐DsRed was increased to 63% (Figs 6A and S4B).

We also studied co‐localization of NbPI4KII‐mGFP with Rep domains Rep_1‐120_‐DsRed, Rep_1‐180_‐DsRed and Rep_181‐361_‐DsRed. We found that Rep_1‐180_‐DsRed and Rep_181‐361_‐DsRed domains are able to draw NbPI4KII‐mGFP into the nucleus, and Rep_1‐180_‐DsRed is able to draw 87% of cells with NbPI4KII‐mGFP exclusively inside the nucleus (*N* = 16) (Figs 6D and S4B). Also, Rep_181‐361_‐DsRed is able to redirect NbPI4KII‐mGFP into the nucleus in 78% of cells (*N* = 17) (Figs 6E and S4B).

Furthermore, we investigated effect of Rep on subcellular localization of CaPI4KII. We observed the expression of CaPI4KII‐mGFP along with Rep‐DsRed. We found that CaPI4KII is present in three states, i.e. cytoplasm and nucleus (81%), cytoplasm (8%) or nucleus (11%), in the presence of Rep‐DsRed (Figs S7B and S5F).

We also performed co‐localization of CaPI4KII‐mGFP with Rep mutants Rep_1‐120_‐DsRed, Rep_1‐180_‐DsRed and Rep_181‐361_‐DsRed. When CaPI4KII‐mGFP was co‐expressed with Rep_1‐120_‐DsRed, 60% of cells showed expression of CaPI4KII‐mGFP inside the cytoplasm and nucleus, and 20% showed expression restricted to the nucleus or cytoplasm (Fig. S7C,F). CaPI4KII‐mGFP, when co‐expressed with Rep_1‐180_‐DsRed, showed 33% of cells with CaPI4KII‐mGFP expression in the cytoplasm and nucleus, 12% of cells showing expression in the cytoplasm and 61% of cells showing expression of CaPI4KII‐mGFP restricted to the nucleus (Fig. S7D,F). When CaPI4KII‐mGFP was co‐expressed with Rep_181‐361_‐DsRed, we found 41% of cells showing expression in both the cytoplasm and nucleus, 17% of cells showing expression in the cytoplasm and 41% of cells showing expression restricted to the nucleus (Fig. S7E,F). This observation suggests that Rep_1‐180_‐DsRed and Rep_181‐361_‐DsRed can redirect CaPI4KII‐mGFP into the nucleus whereas Rep_1‐120_‐DsRed failed to do this (Fig. S7D–F). Therefore, Rep‐DsRed appears to enhance the nuclear occurrence of both NbPI4KII‐mGFP and CaPI4KII‐mGFP into the nucleus.

### Functional characterization of NbPI4KII

**Figure 7:**
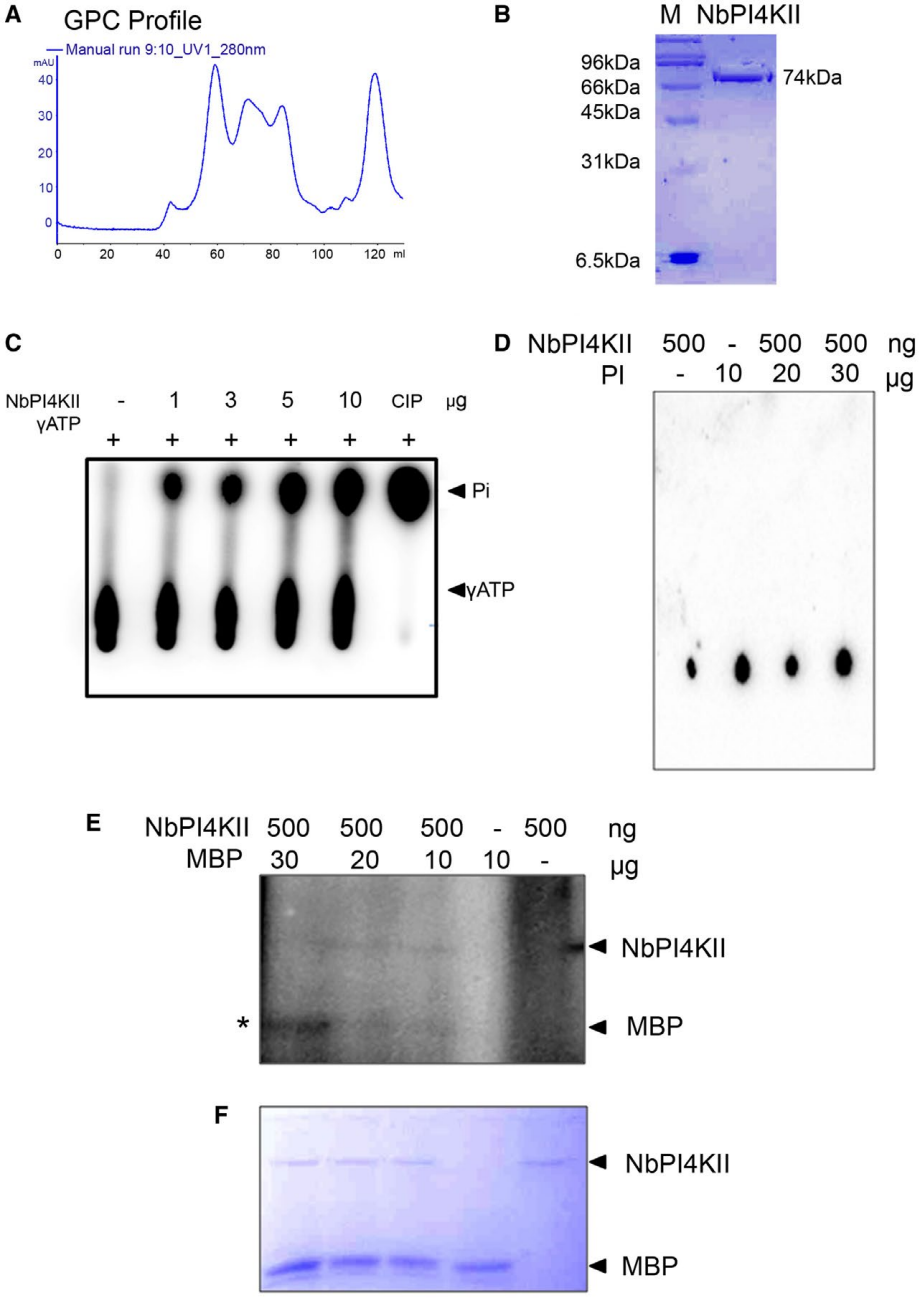
Biochemical characterization of NbPI4KII. (A) Gel permeation chromatography (GPC) of NbPI4KII using Superdex 200G beads. (B) Ni^2+^‐NTA and GPC‐purified NbPI4KII protein run on 10% SDS‐PAGE. (C) ATPase assay of NbPI4KII with varying concentrations ranging from 1 to 10 µg, calf intestine phosphatase (CIP) is used as positive control. (D) Lipid kinase assay of NbPI4KII. Varying concentration of phosphoinositide (PI) ranging from 10 to 30 µg was tested and 500 ng of NbPI4KII was employed in the presence of γ^32^P (ATP). (E) Protein kinase assay of NbPI4KII (500 ng) with varying concentrations of myelin basic protein (MBP) ranging from 10 to 30 µg in the presence of γ^32^P (ATP). Coomassie brilliant blue stained gel of kinase assay. Asterisk (*) represents phosphorylation. mAU denotes milli‐absorbance units.

NbPI4KII was expressed as C‐terminal histidine‐tagged protein (NbPI4KII‐His6×), under a T7 promoter in prokaryotic expression vector pET28a and mobilized into *Escherichia coli* BL21 cells. In gel permeation chromatography (GPC), the peak at 60 ml was collected (Fig. 7A) and run on SDS‐PAGE (Fig. 7B). The purified protein corresponding to 74 kDa was obtained after GPC (Fig. 7B).

To investigate whether the NPI4KII under query is a functional protein, the ATP hydrolysing activity of the protein was investigated. An ATPase assay was performed by incubating NbPI4KII protein in varying concentrations with radioactive γ^32^P(ATP) (Fig. 7C). The resultant thin‐layer chromatography (TLC) plate, after being autoradiographed, showed release of Pi in the presence of NbPI4KII (Fig. 7C). Also, with an increase in the concentration of NbPI4KII, the proportion of released Pi increases. Calf intestinal phosphatase was used as a positive control and showed complete hydrolysis of ATP into Pi (Fig. 7C). Only ATP did not show the release of Pi. The results show that NbPI4KII hydrolyses ATP in a concentration‐dependent manner. Hence, NbPI4KII may use the gamma‐phosphate of ATP to phosphorylate its substrate and the NbPI4KII under investigation is a functional ATPase.

As NbPI4KII belongs to the lipid kinase family, we tested its lipid kinase activity. The two‐step purified NbPI4KII (Ni^2+^‐NTA and gel permeation chromatography) was assessed for the presence of lipid kinase activity. The purified NbPI4KII was incubated with phosphoinositides (PIs) in reaction buffer in the presence of γ^32^P (ATP). The resultant blot showed no appearance of phosphorylated phosphoinositides up the TLC plate (Fig. 7D). The phosphoinositide fails to get phosphorylated even with increasing concentration of NbPI4KII (Fig. 7D). These results show that the NbPI4KII under query does not possess lipid kinase activity as we failed to detect the NbPI4KII‐mediated phosphorylation of phosphoinositides.

Furthermore, NbPI4KII was tested for protein kinase activity by performing an *in vitro* kinase assay. Purified NbPI4KII was incubated with myelin basic protein (MBP) in the presence of radioactive γ^32^P (ATP) (Fig. 7E). Myelin basic protein was used as a substrate because it acts as substrate for a number of protein kinases, such as protein kinase C (PKC), protein kinase A (PKA), calcium/calmodulin‐dependent protein kinase II (CDPKII) and mitogen‐activated protein kinase (MAPK) (Ulmer, 1988). Substrate alone showed no appearance of the band (Fig. 7E), but in presence of NbPI4KII, the band corresponding to MBP is present, implying phosphorylation of MBP by NbPI4KII (Fig. 7E). An upper band is also visible which corresponds to autophosphorylation of NbPI4KII (Fig. 7E). These results suggest that NbPI4KII phosphorylates MBP in a concentration‐dependent manner (Fig. 7E).

### *NbPI4KII* silencing reduces ChiLCV infectivity

**Figure 8:**
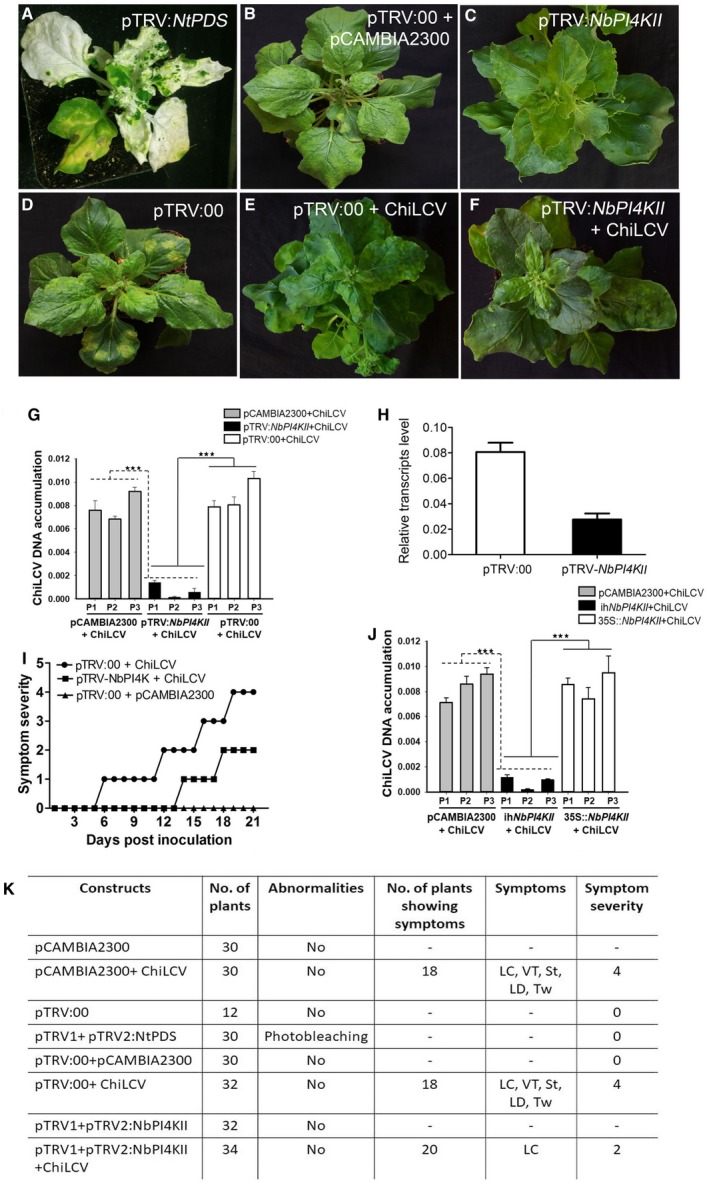
Effect of *NbPI4KII* silencing on ChiLCV pathogenesis. (A) Phytoene desaturase‐silenced representative *Nicotiana benthamiana* plant used as control. (B) Vector control plant (pTRV:00) infiltrated with empty vector pCAMBIA2300. (C) *NbPI4KII‐*silenced plant. (D) Vector control plant (pTRV:00). (E) Vector control plant infiltrated with ChiLCV infectious construct. (F) *NbPI4KII*‐silenced plant infiltrated with ChiLCV infectious construct. (G) Detection of ChiLCV DNA by qPCR in virus‐induced gene silencing (VIGS) plants. (H) Transcript analysis of *NbPI4KII* in pTRV:00 and *NbPI4KII*‐silenced plants. (I) Symptom severity graph of pTRV:00 infiltrated with ChiLCV infectious construct, *NbPI4KII*‐silenced plant infiltrated with ChiLCV infectious construct and pTRV:00 infiltrated with ChiLCV infectious construct. (J) Detection of ChiLCV DNA by qPCR in transiently silenced and overexpressed plants. (K) Table summarizing the effect of *NbPI4KII* VIGS on ChiLCV pathogenesis.

To study the effect of NbPI4KII in ChiLCV pathogenesis tobacco rattle virus (TRV)‐based virus‐induced gene silencing (VIGS) was performed on *N. benthamiana*. *Phytoene desaturase* silencing (pTRV‐*PDS*) was used as a positive control (Fig. 8A). PDS‐silenced plants showed photobleaching in the tissues where silencing of PDS had occurred (Fig. 8A). *Nicotiana benthamiana* plants infiltrated with empty vector exhibited typical symptoms of TRV, i.e. mottling of leaves (Fig. 8D). Initially all VIGS plants exhibited very mild TRV symptoms, such as mottling, which diminished with time. The transcript level of *NbPI4KII* was assessed in pTRV control and *NbPI4KII‐*silenced plants, and *NbPI4KII* was significantly reduced in *NbPI4KII*‐silenced plants. Furthermore, *NbPI4KII* silencing was confirmed by detecting *NbPI4KII*‐specific siRNAs (Fig. S8). We were able to detect *NbPI4KII*‐specific siRNAs only in *NbPI4KII*‐silenced plants and not in pTRV plants (Fig. S8). *NbPI4K*II‐silenced *N. benthamiana* plants exhibited no abnormalities (Fig. 8C). The symptoms of ChiLCV on pTRV‐infiltrated and *NbPI4KII*‐silenced plants were compared. pTRV:00 control plants inoculated with empty vector pCAMBIA2300 exhibited no symptoms (Fig. 8B). pTRV:00‐infiltrated plants infected with ChiLCV showed symptoms of ChiLCV, namely, downward leaf curling and stunting of plants (Fig. 8E). *NbPI4KII*‐silenced plants inoculated with ChiLCV exhibited diminished symptoms compared to pTRV:00 plants inoculated with ChiLCV (Fig. 8F). Furthermore, northern hybridization was carried out to determine the role of NbPI4KII on TRV pathogenesis. Results indicated that TRV titre remained mostly unchanged in plants infiltrated with TRV or TRV‐*NbPI4KII* or TRV‐*NbPI4KII* + ChiLCV infiltrated plants (Fig. S9A). These results suggest that NbPI4KII did not influence TRV accumulation in *N. benthamiana*.

Plants infiltrated with empty vector followed by ChiLCV infiltration showed a maximum symptom severity score of 4 at 21 days post‐infiltration (dpi), whereas in *NbPI4KII*‐silenced plants showed maximum symptom severity of 2 at 21 dpi (Fig. 8I). Also, the onset of symptoms was variable between the control and *NbPI4KII‐*silenced plants infected with ChiLCV. Symptoms started as early as 5 dpi in TRV plants infected with ChiLCV, whereas symptoms started at 13 dpi in *NbPI4KII‐*silenced plants infected with ChiLCV (Fig. 8I). Furthermore, the ChiLCV DNA level was assessed in silenced as well as in mock plants inoculated with ChiLCV. We found that ChiLCV DNA quantities significantly diminished compared to the TRV‐ and ChiLCV‐infiltrated plants (Figs 8J and S9B). Virus DNA was also assessed by qPCR and found to be three‐fold downregulated in *NbPI4KII*‐silenced plants as compared to control plants (Fig. 8G).

Transient *in planta* assays were carried out on *N. benthamiana* plants using CaMV 35S:*NbPI4KII* and *NbPI4KII* RNAi constructs to validate the role of NbPI4KII on ChiLCV pathogenesis. Plants were initially infiltrated with *Agrobacterium tumefaciens* EHA 105 containing the above‐mentioned constructs at the five‐ to six‐leaf stage followed by agroinfiltration of these plants with ChiLCV. qPCR analysis revealed that NbPI4KII‐silenced *N. benthamiana* plants contained a significantly reduced level of ChiLCV titre as compared to plants infiltrated with either CaMV 35S:*NbPI4KII* + ChiLCV or pCAMBIA2300 + ChiLCV (Fig. S9C).

## Discussion

Plant viruses are known to use host protein kinases as they are key regulators of several pathways in the plant cell. Kinases function by switching on or off their targets via phosphorylation. By hijacking host kinase activities, viruses can manipulate the cell environment for its own favour. In an earlier study, expression of PI4K was observed to be differentially regulated in *C. annuum* plants following ChiLCV infection (Kushwaha *et al*., 2015). In the present study, we detected an enhanced level of *PI4KII* in both *N. benthamiana* and *C. annuum* plants inoculated with ChiLCV. Furthermore, *PI4KII* was isolated from *N. benthamiana* as well as *C. annuum* plants. Phylogenetic analysis of sequences revealed that the PI4Ks of this study belong to PI4K type II clade. We found that NbPI4KII interacted with four of the ChiLCV‐encoded proteins: Rep, TrAP, C4 and V2. Phosphatidylinositol 4‐kinase is known to be used by several RNA viruses infecting *H. sapiens*. HCV hijacks PI4KIIIα in order to create its own replication niche and this is achieved as PI4KIIIα produces PI4P at the site of replication, forming a membranous web and the replicating virus is thus shielded from the host defence machinery (Berger *et al*., 2009, 2011). Recently, the role of type II PI4Kγ3 in abiotic stress response and floral transition in plants has been demonstrated (Akhter *et al*., 2016) while another type II PI4K (γ5) interacts with membrane target transcription factor ANAC078 to regulate auxin biosynthesis and leaf margin development (Tang *et al*., 2016). However, the role of PI4Ks in plant virus bi‐fluorescence complementation assay pathogenesis is not yet known.

Previous studies suggested that both PI4KIIIα and PI4KIIIβ are exploited by RNA viruses for successful infection. HCV hijacks PI4KIIIα by interaction of PI4KIIIα with the nonstructural protein NS5A, resulting in recruitment of the former to the replication foci (Lim and Hwang, 2011). PI4KIIIα generates a PI4P membranous web where the virus replication takes place. PI4KIIIβ associates with viral proteins 3A, 3AB, 3CD and 3D of enterovirus. Hence, enterovirus exhibits GBF1/ARF1‐dependent recruitment of PI4KIIIβ for formation of PI4P‐enriched replication complexes. Conversely, Aichi virus recruits PI4KIIIβ with the help of Golgi protein acyl‐coenzyme A binding domain protein 3 (ACBD3), whose primary function is to maintain the structure of the Golgi apparatus. The nonstructural proteins of Aichi virus, i.e. 2B, 2BC, 2C, 3A and 3AB, interact with ACBD3 (Sasaki *et al*., 2012). ACBD3 in turn interacts with PI4KIIIβ (Greninger *et al*., 2012; Sasaki *et al*., 2012). In the present study, the NbPI4KII interacts with the Rep protein of ChiLCV. The physical interaction was found to be direct as confirmed by FRET and BiFC experiments. The interaction of NbPI4KII with Rep was mapped to all the domains of Rep, which indicate that the full‐length Rep protein is involved in the interaction. CaPI4KII full‐length protein failed to interact with Rep. However, we found that CaPI4KII interacted with Rep_1‐180_ and Rep_181‐361_ but not with Rep_1‐120_. There is 80% similarity between NbPI4KII and CaPI4KII, and the difference in their interacting abilities may be attributed to the difference in protein sequences. Additionally, as previously reported, different domains of ChiLCV‐encoded Rep protein are known to perform different functions (Orozco *et al*., 1997) and these domains are differently localized in the host cell (Kushwaha *et al*., 2017). The interaction between different Rep domains and NbPI4KII suggests an ability of this host protein to positively regulate Rep functions through interaction with all Rep domains and hence the infectivity of ChiLCV is enhanced. However, CaPI4KII interacts with the domain of the Rep protein only when it is not engaged into nucleolar functions. It assumes greater importance as oligomerization domains of Rep (20‐80 amino acids) appears to play crucial role in establishing interaction between Rep and CaPI4KII.

Different compartmentalization of PI4K influences the outcomes (Balla *et al*., 2002). Localization of a protein reveals much about the nature of its function. Different isoforms of PI4K revealed a varied pattern of subcellular localization. HsPI4KIIβ from *H. sapiens* localized in the cytosol and also in the perinuclear region and at the plasma membrane in HeLa cells (Wei *et al*., 2002). On the other hand, HsPI4KIIα was localized to Golgi bodies alone (Wei *et al*., 2002). Yeast Pik1 localizes to the nucleus and contributes to the nuclear phosphoinositide pool (Garcia‐Bustos *et al*., 1994). Another yeast homologue of PI4K, Stt4, has been shown to be present at the plasma membrane (Audhya and Emr, 2002). Rice OsUbDKγ4, a homologue of AtPI4Kγ4, localizes to the cytoplasm (ER) and nucleus (Song *et al*., 2017). NbPI4KII or CaPI4KII were found to be present in the cytoplasm and nucleus. The maximum fraction of cells showed occurrence inside both the cytoplasm and nucleus as compared to either cytoplasm‐ or nucleus‐alone. Our study suggests the association of NbPI4KII‐mGFP and CaPI4KII‐mGFP with ER and Golgi bodies in the cytoplasm.

PI4KIIIα also colocalizes with NS5A of HCV at the site of replication of complexes because it produces a pool of PI4P to facilitate a niche for HCV replication (Berger *et al*., 2009). NbPI4KII interacts with more than one viral protein, which appears to be a strategy of the virus to completely hijack the NbPI4KII for successful pathogenesis. The interaction also influences the localization of either NbPI4KII or CaPI4KII, which is another indication of the above‐mentioned host proteins being exploited by the viral Rep protein. Both NbPI4KII and CaPI4KII, when co‐expressed with Rep protein, showed maximum occurrence inside the nucleus with a smaller fraction of cells displaying either NbPI4KII or CaPI4KII in the cytoplasm. We also performed colocalization of either NbPI4KII or CaPI4KII with Rep domains and found the results in accordance with yeast two‐hybrid and BiFC results. This suggests that ChiLCV Rep protein is responsible for enhancing nuclear localization of NbPI4KII or CaPI4KII.

NbPI4KII was found to be a functional protein as it could hydrolyse ATP. *In vitro* lipid kinase activity of NbPI4KII could not be detected. Unlike conventional PI4Ks, NbPI4KII lacks the pleckstrin homology domain that is required for binding to lipids (Harlan *et al*., 1994), which may account for the inability of NbPI4KII to phosphorylate phosphoinositides. Since the cell is already occupied with various homologues of PI4K that contribute to the phosphoinositide pool, PI4K type II has evolved to lose lipid kinase activity. Instead, NbPI4KII possesses protein kinase activity. *Arabidopsis thaliana* type II PI4Ks also exhibited similar protein kinase activity but displayed lack of lipid phosphorylation activity (Galvao *et al*., 2008). It is proposed that NbPI4KII is a member of this new clade of protein kinases lacking lipid kinase activity. Since ChiLCV‐encoded Rep interacts with NbPI4KII, it is also possible that ChiLCV‐encoded Rep is phosphorylated by NbPI4KII, which may favour pathogenesis of ChiLCV. However, further investigations are required to ascertain this.

Transient silencing of *NbPI4KII* led to significant reduction of ChiLCV DNA accumulation. *NbPI4KII‐*silenced plants also exhibited late onset of symptoms, implying that NbPI4KII is required for ChiLCV pathogenesis. *NbPI4KII‐*silenced plants did not manifest any abnormalities other than the TRV symptoms, which indicates that silencing of *NbPI4KII* is not lethal for the plant. This suggests that ChiLCV targets a host protein that is not crucial for the survival of the plant. Rep interacts with NbPI4KII and hijacks it for its own welfare. This is similar to *PI4KIIIα‐*silencing, resulting in significant reduction of HCV viral titre (Berger *et al*., 2009). Utilization of PI4KIIIβ by Aichi virus comes from the evidence that knockdown of PI4KIIIβ by siRNAs retarded viral RNA replication to a significant extent.

In a previous study, HsPI4KIIIα localized to the ER in the presence of HCV (Berger *et al*., 2009). As Rep influences the localization of both NbPI4KII and CaPI4KII by physically associating with these proteins, it was interesting to observe the subcellular localization of either NbPI4KII or CaPI4KII in the presence of the virus. We also found that the expression of *NbPI4KII* or *CaPI4KII* is enhanced in the presence of ChiLCV. Also, both NbPI4KII‐mGFP and CaPI4KII‐mGFP showed association with cytoplasmic organelles like ER and Golgi bodies in the presence of ChiLCV. These results suggest that during ChiLCV pathogenesis PI4KII may be involved in some other functions, probably with coordination with other viral proteins.

ChiLCV‐encoded Rep interacts with NbPI4KII thereby enhancing its accumulation inside the nucleus. Downregulation of *NbPI4KII* reduces viral titre Plausibly, NbPI4KII is a positive regulator of ChiLCV in *N. benthamiana*. To the best of our knowledge this is the first report of involvement of NbPI4KII in modulating plant virus pathogenesis.  In summary, the current study suggests that PI4KII functions as a positive regulator, which modulates ChiLCV pathogenesis through interaction with the viral Rep protein. Identification of PI4K plant targets and the molecular mechanism of PI4K‐mediated geminivirus pathogenesis need to be understood in the future.

## Experimental Procedures

### Construct

The partial tandem repeats infectious clone of *Chilli leaf curl virus* isolate Varanasi (EF190217), *Chilli leaf curl virus* betasatellite (EF190215) (Chattopadhyay *et al*., 2008) were obtained from the Molecular Virology Laboratory, School of Life Sciences, Jawaharlal Nehru University, New Delhi. *Capsicum annuum* phosphatidylinositol 4‐kinase type II (GenBank accession no. MH544652), *N. benthamiana* phosphatidylinositol 4‐kinase type II (GenBank accession no. MH544653).

### ChiLCV infection

*Nicotiana benthamiana* plants were considered for either agroinfection or agroinfiltration of infectious constructs of ChiLCV depending upon the purpose of experiments. Agroinoculation was performed on the *N. benthamiana* plants as described by Chattopadhyay *et al*. (2008). A primary culture of *A. tumefaciens* strain EHA105 harbouring an infectious viral construct was grown in 3 mL LB medium. A secondary culture was initiated into 50 mL LB medium. The primary culture was used as inoculum and grown at 28 °C for 36 h at 220 rpm. The cells were centrifuged at 6000 ***g*** for 5 min and dissolved in sterile double‐distilled water supplemented with 100 mM acetosyringone and agroinfiltrated in *N. benthamiana* plants. The symptoms were monitored and the severity of symptoms was recorded following Chakraborty *et al. *(2008).

### Cloning and phylogenetic and domain analysis of PI4KII

We cloned the full‐length 1.9 kb cDNA from *N. benthamiana and C.* *annuum* using primer pair 5′‐GGATCCATGTCGAGGAACTTAGACAGTCCT‐3′ and 5′‐GTCGACTCAAAACTGGCATGAAGTGCC‐3′ or 5′‐GTCGACATGTCGAGGAACTTAGACAGTCCT‐3′ and 5′‐AGATCTGCATGAAGTGCCAAGTCTCTGT‐3′, respectively, in blunt end vector pJET1.2 (Thermo Fisher Scientific, MA, USA). Amino acid sequences of different PI4Ks isolated from *H. sapiens*, *S. cerevisiae*, *A. thaliana*, *C. annuum*, *S. lycopersicum* and *O. sativa* were extracted from the NCBI database. Evolutionary analyses were conducted using Molecular Evolutionary Genetics Analysis version 7.0 (MEGA7) (Kumar *et al*., 2016). The evolutionary history was inferred by the maximum likelihood method based on the JTT matrix‐based model. The tree with the highest log‐likelihood (–16573.85) is presented. Initial tree(s) for the heuristic search were obtained automatically by applying neighbour‐joining and BioNJ algorithms to a matrix of pairwise distances estimated using a JTT model, and the topology with the superior log‐likelihood value was selected. The tree was drawn to scale, with branch lengths measured by the number of substitutions per site. The analysis involved 30 amino acid sequences. All positions containing gaps and missing data were eliminated. There were a total of 269 positions in the final dataset.

The sequences were retrieved from the NCBI database in FASTA format. The FASTA sequences were then scanned through a motif search tool (https://www.genome.jp/tools/motif/). Both NCBI‐CDD and Pfam Motif libraries were selected as preferred libraries. The cut‐off score was narrowed down to 1.0 for stringency of the search. The data generated using this software were also validated by the interpro (https://www.ebi.ac.uk/interpro/) and Prosite https://prosite.expasy.org/) databases. The domains and motif were identified and subsequently visualized and illustrated by IBS 1.0 software.

### Yeast two‐hybrid assay

*CaPI4KII* and *NbPI4KII* were mobilized in pGDBC1 vector using paired primers 5′‐GGATCCATGTCGAGGAACTTAGACAGTCCT‐3′ and 5′‐GTCGACTCAAAACTGGCATGAAGTGCC‐3′ under *Bam*HI/*Sal*I or 5′‐GTCGACATGTCGAGGAACTTAGACAGTCCT‐3′ and 5′‐AGATCTGCATGAAGTGCCAAGTCTCTGT‐3′ under *Sal*I/*Bgl*II sites, respectively. *Saccharomyces cerevisiae* strain AH109 was transformed using the lithium acetate method following the manufacturer’s instructions (Clonetech, CA, USA). Plating was carried out on two‐drop‐out (2DO) YPDA plates (−leu, −trp). Colonies appearing after 2–3 days were streaked on 3DO plates (−leu, −his, −trp) supplemented with 5 mM 3‐amino‐1,2,4‐triazole (3AT). A growth kinetic study of the interaction between NbPI4KII and Rep full‐length and domains was carried out to assess the strength of each interaction. For this, colonies were grown in 3DO medium supplemented with 10 mM 3AT. The final absorbance of each combination was measured at 600 nm.

### Subcellular localization of PI4Ks

*NbPI4KII* and *CaPI4KII* were cloned in pCAMBIA1304 in single‐site *Bgl*II using primer pair 5′‐AGATCTAATGTCGAGGAACTTAGACAGTCCTGTTCAG‐3′ and 5′‐AGATCTGCATGAAGTGCCAAGTCTCTGT‐3′. *Agrobacterium tumefaciens* strain 2260 harbouring either *NbPI4KII‐* or *CaPI4KII*‐pCAMBIA1304 was allowed to grow in LB medium containing Rifampicin (30 ppm), Kanamycin (50 ppm) and Carbenicillin (50 ppm) for 48 h. Cells were harvested at 6000 ***g*** for 5 min and dissolved in infiltration buffer to obtain OD_600_ = 0.5. The buffer containing *Agrobacterium* was infiltrated in the lower epidermis of *N. benthamiana* plants with a needleless syringe. To observe the subcellular localization of NbPI4KII and CaPI4KII, the lower epidermis was peeled off and stained with DAPI (4 μg/mL) and observed under a microscope (Eclipse TiE, Nikon, Tokyo, Japan).

### Overexpression and purification of NbPI4KII

*NbPI4KII* was cloned in pET28a vector under *Sal*I and *Xho*I sites using primer pair 5′‐GTCGACAAATGTCGAGGAACTTAGACAGT‐3′ and 5′‐CTCGAGTCAAAACTGGCATGAAGTGCC‐3′. The confirmed clone was mobilized into *E. coli* BL21. A single colony of *E. coli* BL21 harbouring *NbPI4KII* was inoculated in LB medium containing kanamycin (50 ppm) and was grown overnight at 37 °C at 220 rpm. The secondary culture was set up by adding 1% of primary culture and grown at 37 °C at 220 rpm till OD_600_ = 0.6. Induction with 0.1 mM IPTG was given at OD_600_ = 0.6 and grown at 12 °C at 220 rpm overnight. The culture obtained was centrifuged at 8000 ***g*** for 5 min at 4 °C. The cells were dissolved in the lysis buffer containing 50 mM Tris‐Cl pH 8.0, 10% glycerol, 300 mM NaCl, 4 mM β mercaptoethanol and 1 mM PMSF. Lysozyme (0.1 mg/mL) was supplemented to lyse the cells. Sonication was performed with 10 cycles of 10 s on and 40 s off at 25% amplitude. The sonicated sample was clarified at 16 000 ***g*** for 30 min. The Ni^2+^‐NTA column was equilibrated with 50 mM Tris pH 8.0, 300 mM NaCl and 10% glycerol followed by binding of the supernatant. The washing was done with 10 mM lysis buffer containing 10 mM imidazole. Protein elution was carried out with 300 mM imidazole. Laemmli buffer (1×) (Laemmli *et al*., 1970) was added to the eluted samples and denatured at 100 °C for 10 min and run on 10% SDS‐PAGE.

### Gel permeation chromatography

The Ni^2+^‐NTA purified protein was concentrated to 1 mL. The concentrated protein was injected into HiLoadSuperdex 200G (Sigma, St Louis, MO, USA). The protein was run at a speed of 0.8 mL per min. The peak corresponding to 40 mAU at 60 mL was collected and run on SDS‐PAGE.

### ATPase assays

The purified NbPI4K_6×His_ was subjected to ATPase assay. ATPase assays were performed as described previously (George *et al*., 2014). The proteins at varying concentrations were incubated with 1:5 dilution of radioactive (γ‐^32^P) ATP in a buffer containing 20 mM Tris ± HCl (pH 8.0), 1 mM MgCl_2_, 100 mM KCl, 8 mM DTT and 80 μg/mL BSA at 25 °C for 15 min. The reaction mixtures were subsequently loaded onto TLC (Sigma) plates and kept in a running buffer (0.5 M LiCl and 1 M methanoic acid). The TLC plates were then air‐dried and autoradiographed.

### Lipid kinase assays

The two‐step purified NbPI4K_6×His_ was subjected to lipid kinase assay. The purified NbPI4K_6__×His _was incubated with phosphoinositides (79403, Sigma) in reaction buffer [10 mM HEPES, 25 mM MgCl_2_, 1 mM EDTA, 1 µCi γ‐^32^P (ATP)] at 25 °C for 15 min. An equal amount of chloroform was added and samples were vigorously vortexed. The samples were centrifuged at 16000 ***g*** for 20 min. The upper aqueous layer was discarded and a lower layer containing lipids was isolated. The isolated lipids were loaded onto TLC plates and run in a chamber saturated with running buffer containing chloroform:methanol:water:7.7 N NH_4_OH in 60:47:11.3:2 ratio until the solvent front reached the top. The TLC plate was dried and kept in the radioactive sensitive plate. The radioactive sensitive plate was scanned using a phosphorimager (Typhoon 9210; Amersham, Little Chalfont, UK) for 12 h.

### Protein kinase assays

Protein kinase assay was performed using NbPI4K_6×His _(500 ng) as enzyme and MBP as a substrate in varying concentrations ranging from 10 to 30 µg. Both the proteins were incubated in reaction buffer containing 10 mM HEPES, 50 mM KCl, 3 mM MgCl_2_, 1 mM DTT, 2 mM CaCl_2_ and 1 µCi γ‐^32^P (ATP) at 37 °C for 30 min. The phosphorylation reaction was terminated by adding Laemmeli buffer and proteins were denatured at 100 °C for 10 min. The reactions were loaded on 10% SDS‐PAGE. The experiment was performed in duplicate. One set of reactions run on SDS‐PAGE were stained with CBB and another set was exposed to a radiosensitive plate for 12 h and scanned on a phosphorimager (Typhoon 9210).

### Fluorescence resonance energy transfer

*Nicotiana benthamiana* plants were infiltrated at the four‐ to five‐leaf stage with *A. tumefaciens* harbouring *NbPI4K*‐*mGFP* in combination with Ds‐Red, Rep‐DsRed, Rep_1‐120_‐DsRed, Rep_1‐180_‐DsRed or Rep_181‐361_‐DsRed. The mGFP and Rep‐DSRed combination was also infiltrated for FRET analysis. *In vivo* expression was monitored at 3 dpi under an Olympus FlluoView FV1000 (Olympus, Tokyo, Japan) and was subjected to FRET. In FRET mGFP served as a donor which transfers energy to DsRed acceptor molecule. FRET was performed as described in Kushwaha *et al *(2017).

### Bimolecular fluorescence complementation assays

Bimolecular fluorescence complementation assay (BiFC) was performed using pSPYNE, and pSPYCE vectors were obtained from the Arabidopsis Biological Resource Center. The ChiLCV ORF C1 was amplified using forward primer 5′‐GGTACCGCCATCGATTTGGAAAACTCC‐3′ and reverse primer 5′‐GGTACCATAAACCTCCAACGGAGGTG‐3′, and cloned into the pSPYCE vector at *Kpn*I and *Xho*I sites. Similarly, *NbPI4KII* was amplified using forward primer 5′‐GTCGACATGTCGAGGAACTTAGACAGT‐3′ and reverse primer: 5′‐CCCGGGTCAAAACTGGCATGAAGTGCC‐3′, and cloned into the pSPYNE vector at the *Sal*I and *Xma*I sites. The pSCPYCE‐Rep, pSPYNE‐*NbPI4KII* constructs were transformed into *A*. *tumefaciens* strain GV2260. BiFC assay was done by co‐infiltration of pSPYCE‐REP with pSPYNE‐*NbPI4KII* into the lower epidermis of *N*. *benthamiana* following Kushwaha *et al. *(2017). *In vivo* expression was observed under a confocal microscope (Model Eclipse TiE, Nikon, Tokyo, Japan). NIS‐Element 4.0 software (Nikon, Tokyo, Japan) was also used to subtract the background signals.

### Virus‐induced gene silencing

A tobacco rattle virus (TRV)‐based vector was employed to silence *NbPI4KII* in *N*. *benthamiana*. pTRV1 (CD3‐1039), pTRV2 (CD3‐1040) and pTRV2‐*PDS* (CD3‐1045) vectors procured from the Arabidopsis Biological Resource Center. The N‐terminal 400 bp of *NbPI4KII* was amplified using primer pair FP 5′‐ CTCGAGGTCGAGGAACTTAGACAGTCCTG‐3′ and RP 5′‐GGATCCTTCCCAGTATTTCAATGGG‐3′, and mobilized into the pTRV2 vector between the enzymes *Sac*I and *Bam*HI sites. *Nicotiana benthamiana* plants at the three‐ to four‐leaf stage were infiltrated with *A.* *tumefaciens* GV2260 harbouring pTRV1 along with pTRV2‐*NbPI4KII*. Plants infiltrated with pTRV1 and pTRV2 served as control. At 10 dpi, new (systemic) leaves of *N. benthamiana* plants were infiltrated with *A.* *tumefaciens* GV2260 harbouring either ChiLCV or pCAMBIA2300. The symptom severity of ChiLCV was monitored during the course of infection. Samples were collected at 21 dpi of ChiLCV infection. VIGS was performed twice with triplicate samples each time.

### Transient *in planta* silencing and overexpression assays

For transient assays, overexpression and RNAi constructs of NbPI4KII were used for infiltration of *N. benthamiana* plants. The *NbPI4KII* RNAi construct (ihNbPI4KII) was generated by cloning the 344 bp segment of the 3′‐UTR of the gene in an inverted orientation separated by the NtPDS intron. The forward arm (344 bp) was amplified by the primer pairs FP 5′‐CTCGAGGCAAAGGCTTGGGACTT‐3′ and RP 5′‐GAGCTCTT TTCATATTTCATTCTAC‐3′, while the reverse arm was amplified by the primers FP 5′‐GGTACCTTTTCATATTTCATTCTAC‐3′ and RP 5′‐GGATCCGCAAAGGTTGGGACTT‐3′. The *NtPDS* intron (700 bp) was PCR amplified using primer pairs FP 5′‐GAGCTCGTAAGTTCTCACTGGTTGT‐3′ and RP 5′‐GGTACCCTGCAAATATAGGTGTATA‐3′, and the entire cassette was cloned into pCAMBIA2300. For overexpression, the previously described NbPI4KII‐mGFP construct was used whereas pCAMBIA2300 served as control. At first, *N. benthamiana* plants at the five‐ to six‐leaf stage were infiltrated through *A. tumefaciens* harbouring these constructs at the abaxial side followed by reinfiltration with ChiLCV 48 hpi. The uppermost leaves were collected after 15 dpi and viral titre was analysed by both qPCR using ChiLCV AC2/AC3 primers and semiquantitative PCR using ChiLCV AC1‐specific primers.

### Isolation and detection of siRNAs

Total RNA was isolated from the uppermost expanding leaves from either pTRV:00 or pTRV1 + pTRV2‐NbPI4KII *N. benthamiana* plants using Trizol reagent following the manufacturer’s protocol (Sigma). Small RNAs were isolated following Lu *et al*. (2007). *NbPI4KII‐*specific siRNAs were detected using (α‐^32^P dCTP)‐labelled (NbPI4K nt 1‐400) DNA probes. The hybridization was carried out at 42 °C overnight in hybridization buffer (7% SDS, 0.5 M sodium phosphate, 1 mM EDTA). The membrane was washed twice with buffer (2× SSC and 0.2% SDS). The radioactive signal was scanned using a phosphorimager (Typhoon 9210; Amersham, GE Healthcare).

### Isolation of total genomic DNA from plants

Total genomic DNA from the topmost leaves of either virus‐infected or mock‐infiltrated plants was isolated following Dellaporta *et al*. (1983).

### Southern hybridization

Viral DNA accumulation in plant samples was detected by Southern hybridization (Southern, 1975). Southern blot hybridization experiments were carried out according to the protocol described by Sambrook and Russell (2001). Ten micrograms of genomic DNA were electrophoresed in 0.8% agarose gel and transferred to a nylon membrane (positively charged) (MDI Membrane Technologies, Ahmedabad, India). Viral DNA was hybridized with full‐length AC1‐specific radiolabelled (α‐^32^P dCTP) DNA probes. The radioactive signal was imaged using a phosphorimager scanning system (Typhoon 9210, Amersham, GE Healthcare).

### Quantitative real‐time PCR

Expression of *NbPI4KII* and *CaPI4KII* was analysed with the help of qRT‐PCR using primer pair FP 5′‐TAGACGAAAGTAATGAGG‐3′ and RP 5′‐TAAGGTGGTATTCTTGAGAGAC‐3′. Primers for the expression analysis of *PI4KII* were designed using Primer Express v. 3.0 software (Applied Biosystem, CA, USA). RT‐PCR was carried out on an Eco‐Real Time PCR system (Illumina, CA, USA) following Kushwaha *et al*., (2015). 2^‐Δ(ΔCT)^ values from mock‐ and virus‐inoculated plants were plotted using GraphPadPrism 5.0 software (https://www.graphpad.com/scientific-software/prism/). Statistical analysis was performed by the Student’s *t*‐test. Actin expression was used as the internal control. Actin was amplified using the primer pair FP 5′‐GAAGCTCAATCCAAACGTGGTATT‐3′ and RP 5′‐CTCAAACATGATTTGTGTCATC‐3′. To ensure that the PCR product was derived from the mRNA only, parallel reactions lacking cDNA template (NTC) were also performed.

### Detection of TRV transcripts by northern hybridization

Total RNA was isolated from systemic leaves (1 g) using Trizol reagent following the manufacturer’s protocol (Sigma). Twenty micrograms of RNA were separated by 1.2% denaturing agarose gel and transferred onto a positively charged nylon‐66 membrane (MDI). For the detection of tobacco rattle virus (TRV), coat protein (CP) gene of TRV (615 bp) was amplified by PCR using CP‐specific primers and (α^32^P dCTP)‐labelled specific DNA probes. Hybridization was carried out overnight at 60 °C in hybridization buffer (1% SDS, 0.5 M sodium phosphate, 1 mM EDTA). After two washings with buffer (2× SSC and 0.2% SDS), the image was scanned using a phosphorimager (Typhoon 9210).

## Authors Contributions

Mansi, NKK and SC planned and designed the research; Mansi, NKK, AKS and MJK performed the research; Mansi, NKK, AKS, MJK and SC analysed and interpreted data; Mansi, NKK AKS, MJK and SC wrote the manuscript.

## Supporting information


**Fig. S1** Expression analysis of *PI4KII *in infected *N. benthamiana *and *C. annuum *'Punjab lal'.

**Fig. S2** (A) Interaction of ChiLCV proteins with NbPI4KII. (B) Growth kinetic study of yeast cells co‐transformed with NbPI4KII and Rep protein.

**Fig. S3** Interaction between ChiLCV Rep protein and CaPI4KII.

**Fig. S4** Graphical representation of subcellular localization of NbPI4KII‐mGFP in the absence (A) and presence of Rep (B).

**Fig. S5** Subcellular localization of CaPI4KII‐mGFP in lower epidermal cells of *N. benthamiana* leaves.

**Fig. S6** Subcellular localization of CaPI4KII‐mGFP in the presence of ChiLCV.

**Fig. S7** Colocalization study of CaPI4KII‐mGFP with Rep‐DsRed and its mutants.

**Fig. S8** Detection of* NbPI4KII‐*specific siRNAs in pTRV or *NbPI4KII*‐silenced *N. benthamiana* plants.

**Fig. S9** Detection of TRV and ChiLCV in transiently silenced plants.

**Table S1** Percentage identity between amino acid sequences of type II PI4Ks originating from diverse organisms.
