# Corrigendum to “*Nicotiana benthamiana* phosphatidylinositol 4‐kinase type II regulates chilli leaf curl virus pathogenesis”

**DOI:** 10.1111/mpp.13421

**Published:** 2024-01-19

Mansi, Kushwaha, N.K., Singh, A.K., Karim, M.J. and Chakraborty, S. (2019) *Nicotiana benthamiana* phosphatidylinositol 4‐kinase type II regulates chilli leaf curl virus pathogenesis. *Molecular Plant Pathology*, 20(10), 1408–1424. https://doi.org/10.1111/mpp.12846.

In the published version of this article in Figure 6, panels (d) and (e) were mistakenly used for both the NbPI4KII‐mGFP + Rep1‐180‐DsRed and NbPI4KII‐mGFP + Rep181‐361‐DsRed images. The two panels showing individual microscopy images with the NbPI4KII‐mGFP + Rep1‐180‐DsRed and NbPI4KII‐mGFP + Rep181‐361‐DsRed are highly identical; hence this mistake happened during figure assembly. This inadvertent figure error does not in any way alter the interpretations or the conclusions drawn in the manuscript. The corrected Figure 6 is shown below.

**FIGURE 6:**
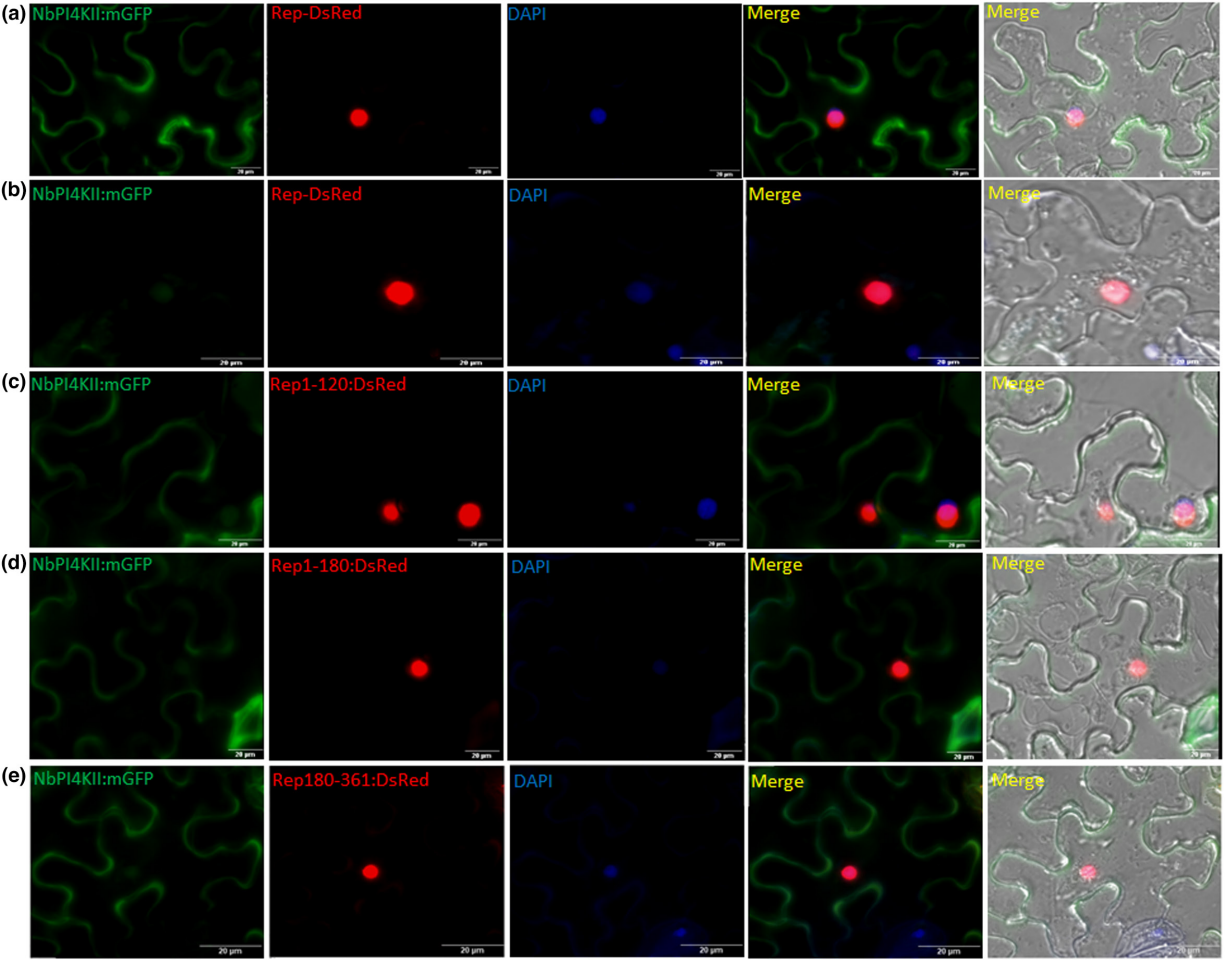
Colocalization study of NbPI4K‐mGFP with Rep‐DsRed and its mutant. Localization of NbPI4KII:mGFP with (a, b) Rep‐DsRed, (c) Rep_1‐120_‐DsRed, (d) Rep_1‐180_‐DsRed and (e) Rep_181‐361_‐DsRed. Scale bar = 20 μm.

We apologize for this error.

