# Supplementary figures and images for: Nicotiana benthamiana phosphatidylinositol 4‐kinase type II regulates chilli leaf curl virus pathogenesis
Source: Mol Plant Pathol. 2019 Sep 2;20(10):1408–24. doi: 10.1111/mpp.12846 (PMC6792133; doi:10.1111/mpp.12846)

**Figure S1**

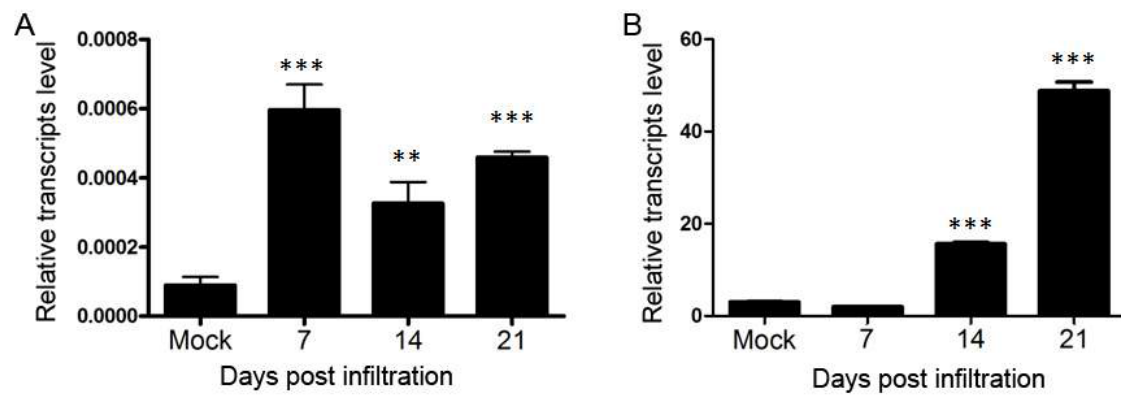

Supplement: Supplementary file 1 — Fig. S1 Expression analysis of PI4KII in infected N. benthamiana and C. annuum 'Punjab lal'. [file MPP-20-1408-s001.pdf]

Figure S2

A)

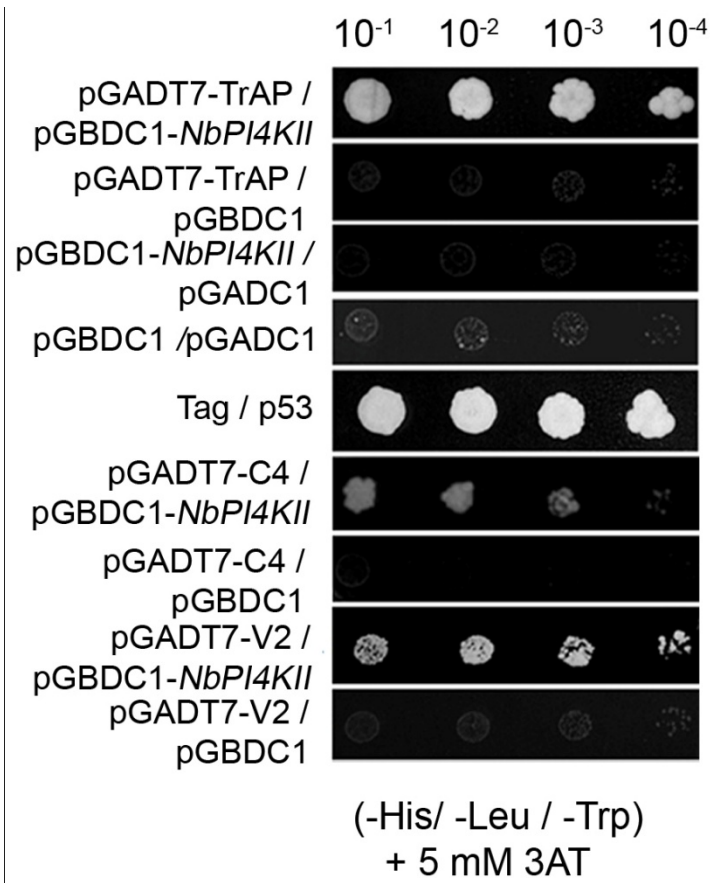

B)

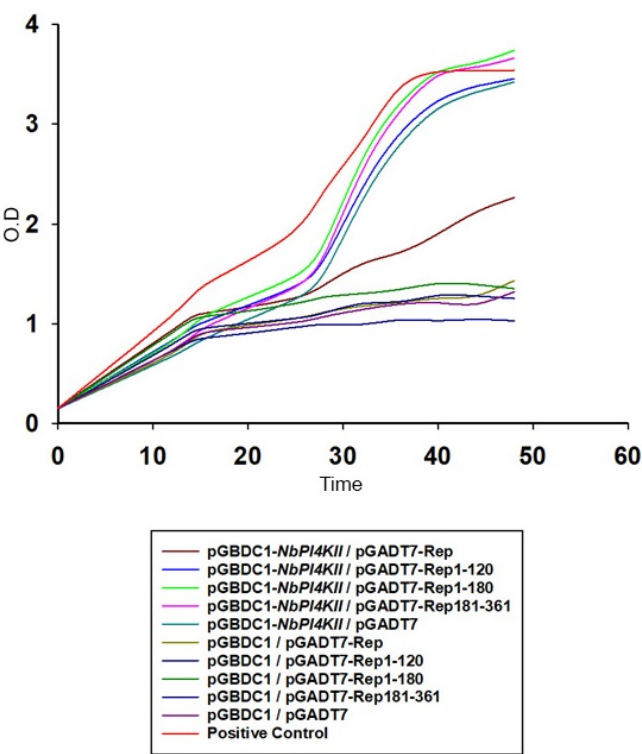

Supplement: Supplementary file 2 — Fig. S2 (A) Interaction of ChiLCV proteins with NbPI4KII. (B) Growth kinetic study of yeast cells co‐transformed with NbPI4KII and Rep protein. [file MPP-20-1408-s002.pdf]

**Figure S3**

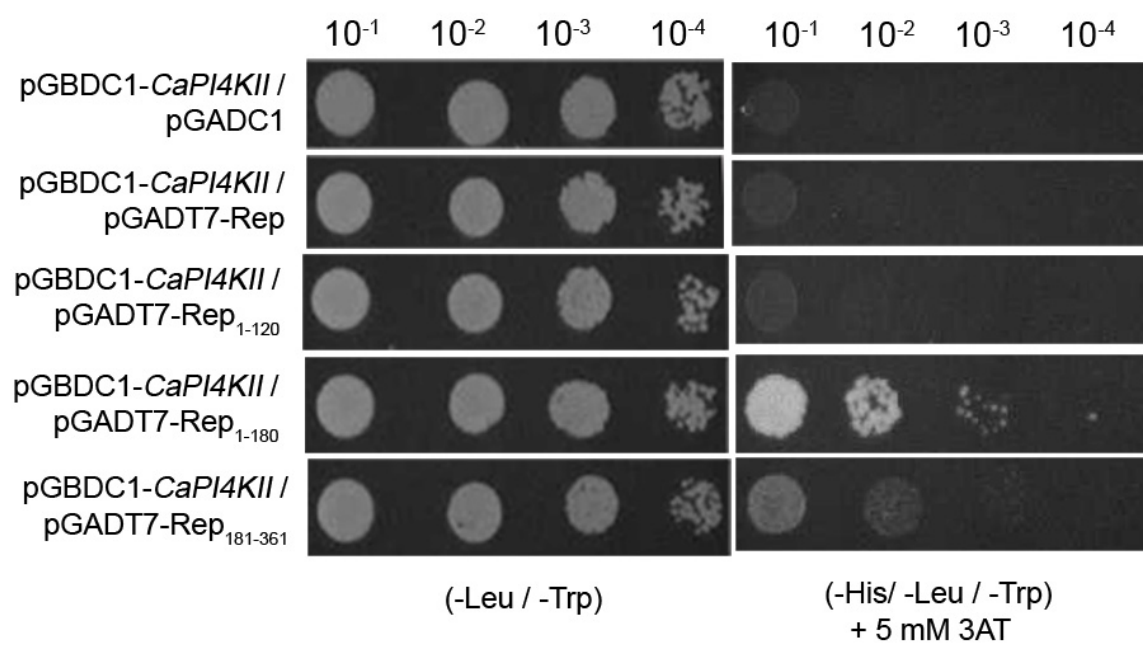

Supplement: Supplementary file 3 — Fig. S3 Interaction between ChiLCV Rep protein and CaPI4KII. [file MPP-20-1408-s003.pdf]

**Figure S4**

**A)**

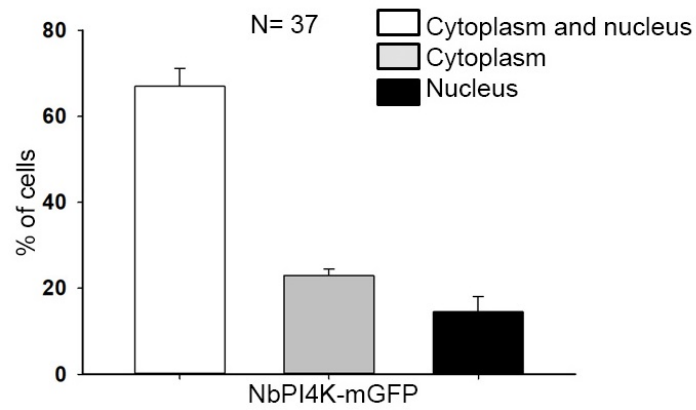

**B)**

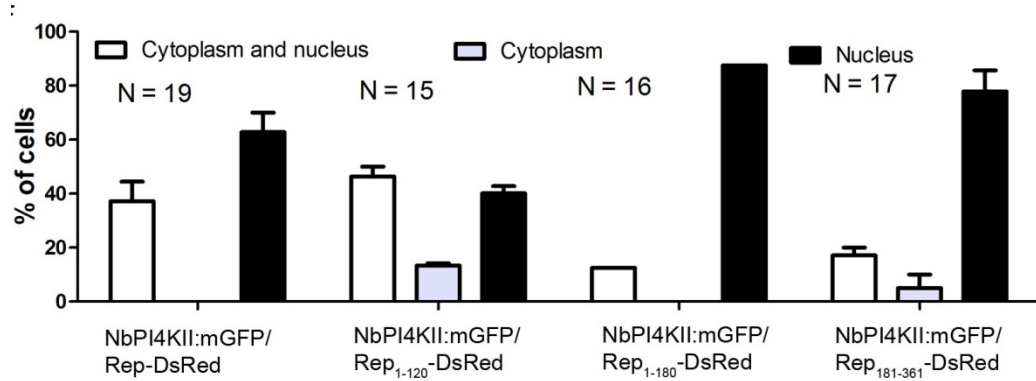

Supplement: Supplementary file 4 — Fig. S4 Graphical representation of subcellular localization of NbPI4KII‐mGFP in the absence (A) and presence of Rep (B). [file MPP-20-1408-s004.pdf]

Figure S5

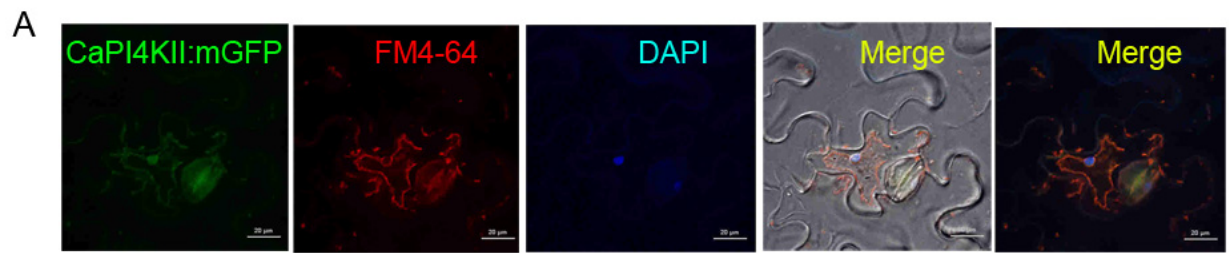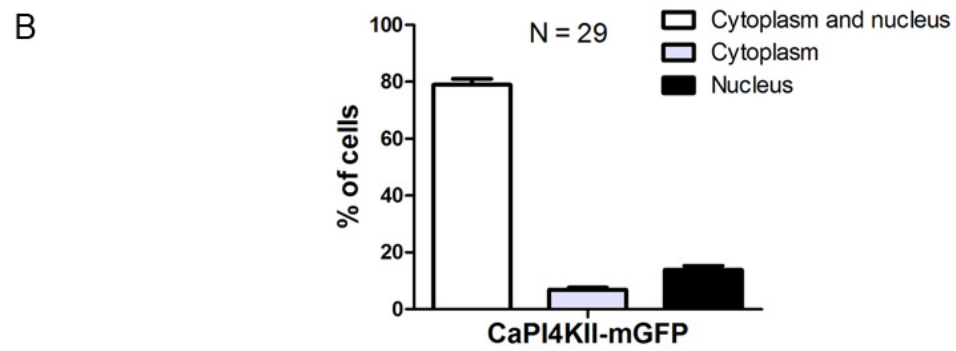

Supplement: Supplementary file 5 — Fig. S5 Subcellular localization of CaPI4KII‐mGFP in lower epidermal cells of N. benthamiana leaves. [file MPP-20-1408-s005.pdf]

**Figure S6**

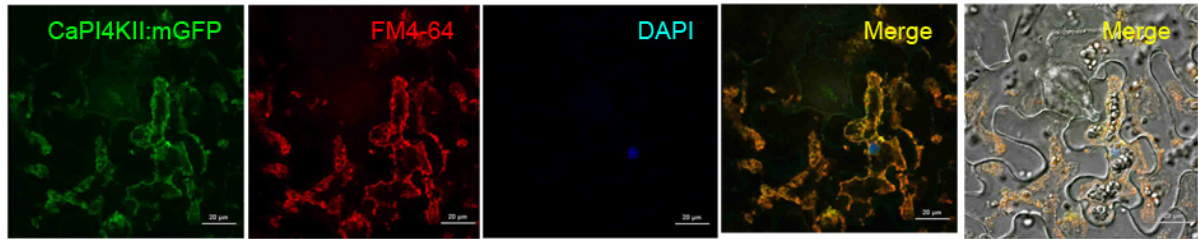

Supplement: Supplementary file 6 — Fig. S6 Subcellular localization of CaPI4KII‐mGFP in the presence of ChiLCV. [file MPP-20-1408-s006.pdf]

Figure S7

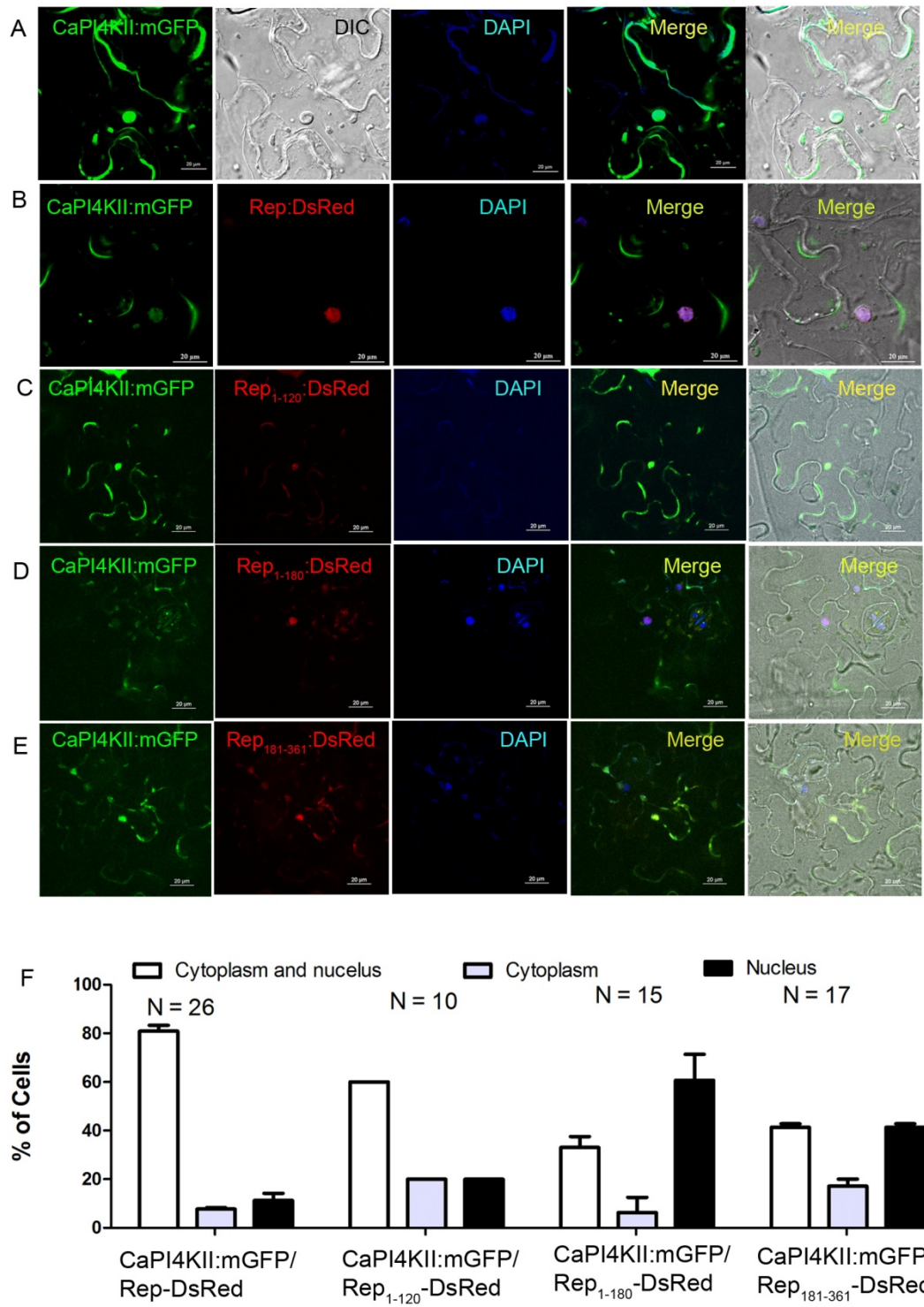

Supplement: Supplementary file 7 — Fig. S7 Colocalization study of CaPI4KII‐mGFP with Rep‐DsRed and its mutants. [file MPP-20-1408-s007.pdf]

**Figure S8**

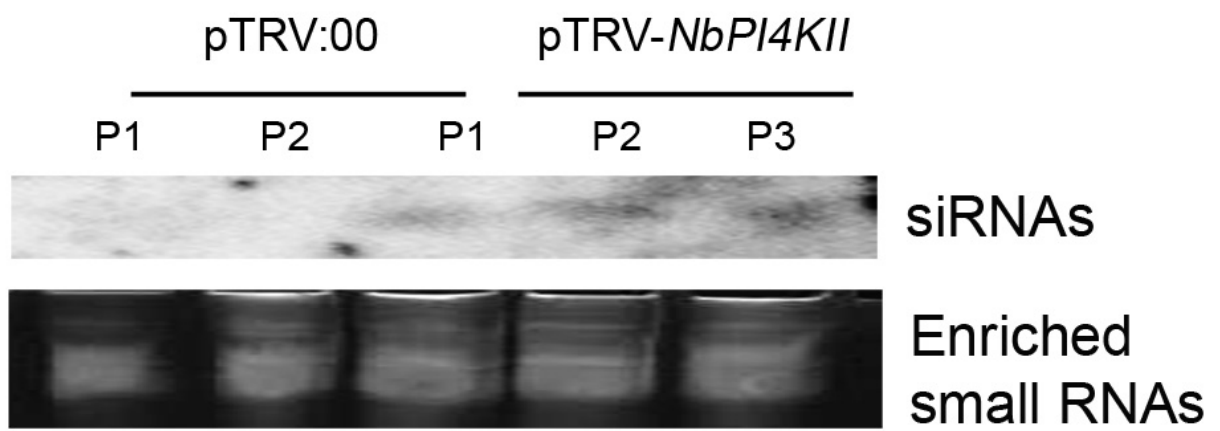

Supplement: Supplementary file 8 — Fig. S8 Detection of NbPI4KII‐specific siRNAs in pTRV or NbPI4KII‐silenced N. benthamiana plants. [file MPP-20-1408-s008.pdf]

Figure S9

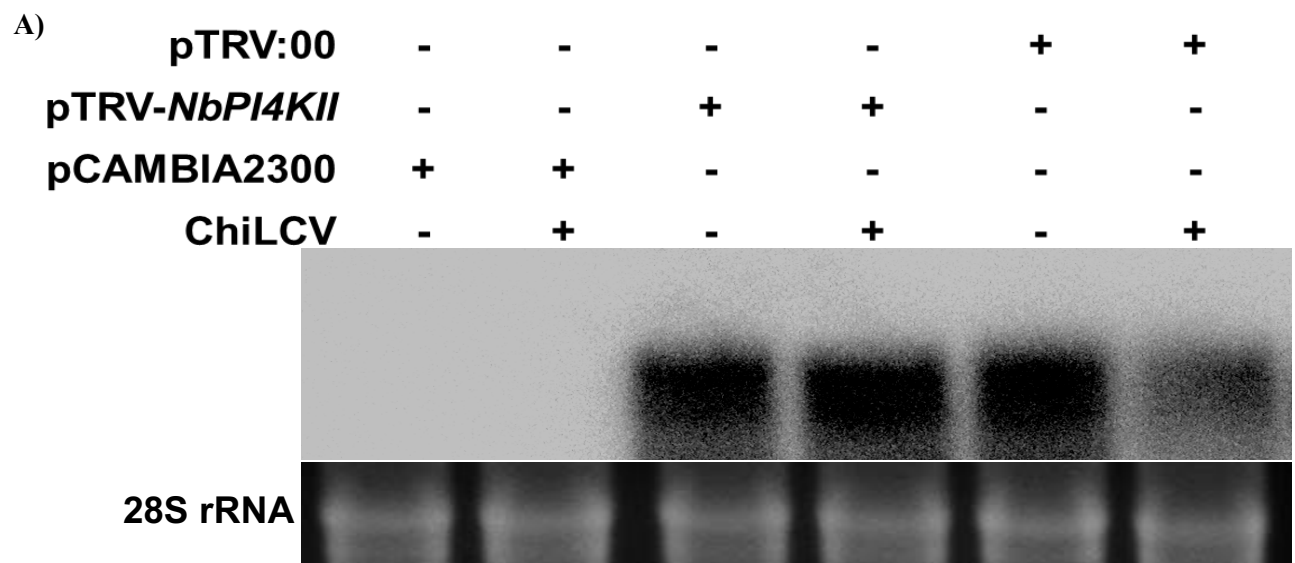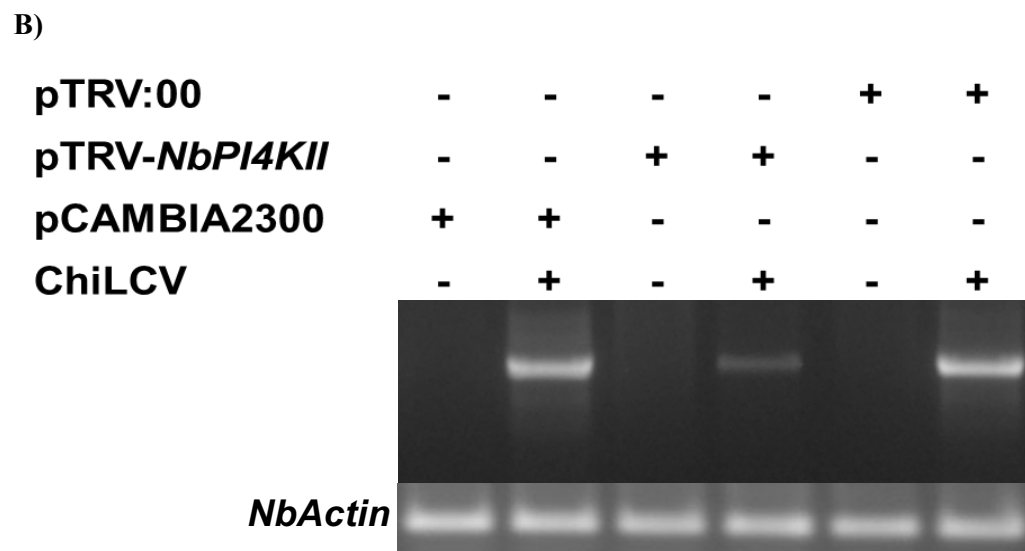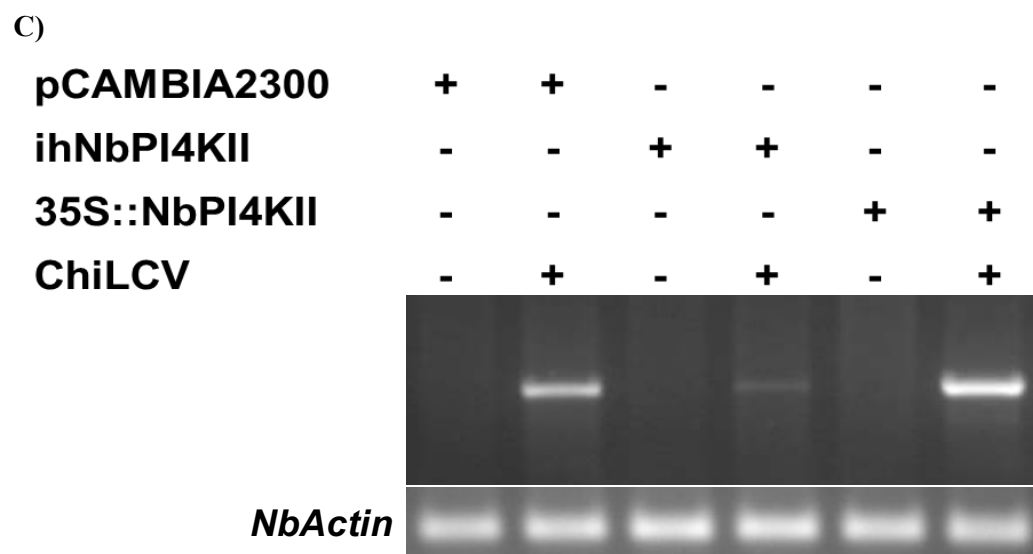

Supplement: Supplementary file 9 — Fig. S9 Detection of TRV and ChiLCV in transiently silenced plants. [file MPP-20-1408-s009.pdf]
