# Supplementary material for: Nicotiana benthamiana phosphatidylinositol 4‐kinase type II regulates chilli leaf curl virus pathogenesis
Source: Mol Plant Pathol. 2019 Sep 2;20(10):1408–24. doi: 10.1111/mpp.12846 (PMC6792133; doi:10.1111/mpp.12846)
Supplement: Supplementary file 10 — Table S1 Percentage identity between amino acid sequences of type II PI4Ks originating from diverse organisms. [file MPP-20-1408-s010.docx]

**Table S1**: Per cent identity between amino acid sequences of Type II PI4Ks originating from diverse organisms

| **Type II PI4Ks** | **HsPI4K2β**  **(Q8TCG2)** | **HsPI4K2α**  **(Q9BTU6.1)** | **ScPI4KLSB6**  **(P42951.1)** | **AtPI4Kγ4**  **(NP_566076.1)** | **AtPI4Kγ2**  **(Q9SGW8)** | **AtPI4Kγ3**  **(Q9FNF8)** | **AtPI4Kγ1**  **(O22199)** | **AtPI4Kγ2like**  **Protein**  **(A0A1I9LRU9)** | **AtPI4Kγ5**  **(Q9C671)** | **AtPI4Kγ6**  **(Q8W4R8)** | **AtPI4Kγ7**  **(Q9SI52)** | [**NbPI4K Type 2**](https://www.ncbi.nlm.nih.gov/nuccore/MH544653.1/)  **(AXU38887.1** | **CaPI4K Type2**  **(AXU38886.1)** | **Sl PI4K Gama 7 (XP_00423901)** |
| --- | --- | --- | --- | --- | --- | --- | --- | --- | --- | --- | --- | --- | --- | --- |
| **HsPI4K2β**  **(Q8TCG2)** | 100 | 57.7 | 24.6 | 15.5 | 15.9 | 19.9 | 16.8 | 14.7 | 18.2 | 16.6 | 14.2 | 15.7 | 14.7 | 17.5 |
| **HsPI4K2α (Q9BTU6.1)** | 57.7 | 100 | 27 | 14.9 | 15.1 | 18.3 | 14.6 | 14.7 | 13.1 | 16.9 | 14.1 | 12.8 | 12.7 | 16.1 |
| **ScPI4KLSB6 (P42951.1)** | 24.6 | 27 | 100 | 14.5 | 13.7 | 14.4 | 18.0 | 19.1 | 17.2 | 15.8 | 14.3 | 16.2 | 16.6 | 17.5 |
| **AtPI4Kγ4 (NP_566076.1)** | 15.5 | 14.9 | 14.5 | 100 | 55.9 | 55.1 | 19.8 | 18.6 | 22.2 | 23.4 | 22.8 | 22.4 | 22.1 | 23.6 |
| **AtPI4Kγ2 (Q9SGW)** | 15.9 | 15.1 | 13.7 | 55.9 | 100 | 50.9 | 20.9 | 20.7 | 22.7 | 21.6 | 22.7 | 22.1 | 22.5 | 24.9 |
| **AtPI4Kγ3**  **(Q9FNF8)** | 19.9 | 18.3 | 14.4 | 55.1 | 50.9 | 100 | 19.9 | 19.3 | 22.5 | 23.4 | 22.1 | 24.1 | 21.8 | 24.8 |
| **AtPI4Kγ1**  **(O22199)** | 16.8 | 14.6 | 18.0 | 19.8 | 20.9 | 19.9 | 100 | 63.6 | 37.7 | 36.3 | 34.9 | 34.2 | 34.5 | 37.1 |
| **AtPI4Kγ2like protein (A0A1I9LRU9)** | 14.7 | 14.7 | 19.1 | 18.6 | 20.7 | 19.3 | 63.6 | 100 | 38.6 | 39.2 | 37.6 | 36.7 | 36.3 | 37.1 |
| **AtPI4Kγ5**  **(Q9C671)** | 18.2 | 13.1 | 17.2 | 22.2 | 22.7 | 22.5 | 37.7 | 38.6 | 100 | 62.7 | 66.1 | 67.9 | 68.1 | 70 |
| **AtPI4Kγ6(Q8W4R8)** | 16.6 | 16.9 | 15.8 | 23.4 | 21.6 | 23.4 | 36.3 | 39.2 | 62.7 | 100 | 74.5 | 66.3 | 65.2 | 67.1 |
| **AtPI4Kγ7**  **(Q9SI52)** | 14.2 | 14.1 | 14.3 | 22.8 | 22.7 | 22.1 | 34.9 | 37.6 | 66.1 | 74.5 | 100 | 70.7 | 68.0 | 71.6 |
| [**NbPI4K Type 2**](https://www.ncbi.nlm.nih.gov/nuccore/MH544653.1/) **(AXU38887.1)** | 15.7 | **12.8** | 16.2 | 22.4 | 22.1 | 24.1 | 34.2 | 36.7 | 67.9 | 66.3 | 70.7 | 100 | 81.8 | **91.4** |
| **CaPI4K Type2(AXU38886)** | 14.7 | **12.7** | 16.6 | 22.1 | 22.5 | 21.8 | 34.5 | 36.3 | 68.1 | 65.2 | 68.0 | 81.8 | 100 | **81.9** |
| **Sl PI4K Gama 7 (XP_00423901)** | 17.5 | 16.1 | 17.5 | 23.6 | 24.9 | 24.8 | 37.1 | 37.1 | 70 | 67.1 | 71.6 | 91.4 | 81.9 | 100 |

Gen Bank accession numbers of each type II PI4K are mentioned in parentheses. Red colured value indicates minimum identity whereas blue coloured indicates maximum identity.
